# Diffusion MRI with pulsed and free gradient waveforms: Effects of restricted diffusion and exchange

**DOI:** 10.1002/nbm.4827

**Published:** 2022-09-27

**Authors:** Arthur Chakwizira, Carl‐Fredrik Westin, Jan Brabec, Samo Lasič, Linda Knutsson, Filip Szczepankiewicz, Markus Nilsson

**Affiliations:** ^1^ Department of Medical Radiation Physics, Lund Lund University Lund Sweden; ^2^ Department of Radiology, Brigham and Women's Hospital Harvard Medical School Boston Massachusetts USA; ^3^ Danish Research Centre for Magnetic Resonance, Centre for Functional and Diagnostic Imaging and Research Copenhagen University Hospital ‐ Amager and Hvidovre Copenhagen Denmark; ^4^ Random Walk Imaging AB Lund Sweden; ^5^ Russell H. Morgan Department of Radiology and Radiological Science Johns Hopkins University School of Medicine Baltimore Maryland USA; ^6^ F. M. Kirby Research Center for Functional Brain Imaging Kennedy Krieger Institute Baltimore Maryland USA; ^7^ Department of Clinical Sciences Lund, Radiology Lund University Lund Sweden

**Keywords:** cumulant expansion, diffusion MRI, exchange, gradient waveform, restricted diffusion, restriction‐exchange space, restriction‐exchange weighting, time dependence

## Abstract

Monitoring time dependence with diffusion MRI yields observables sensitive to compartment sizes (restricted diffusion) and membrane permeability (water exchange). However, restricted diffusion and exchange have opposite effects on the diffusion‐weighted signal, which can lead to errors in parameter estimates. In this work, we propose a signal representation that incorporates the effects of both restricted diffusion and exchange up to second order in b‐value and is compatible with gradient waveforms of arbitrary shape. The representation features mappings from a gradient waveform to two scalars that separately control the sensitivity to restriction and exchange. We demonstrate that these scalars span a two‐dimensional space that can be used to choose waveforms that selectively probe restricted diffusion or exchange, eliminating the correlation between the two phenomena. We found that waveforms with specific but unconventional shapes provide an advantage over conventional pulsed and oscillating gradient acquisitions. We also show that parametrization of waveforms into a two‐dimensional space can be used to understand protocols from other approaches that probe restricted diffusion and exchange. For example, we found that the variation of mixing time in filter‐exchange imaging corresponds to variation of our exchange‐weighting scalar at a fixed value of the restriction‐weighting scalar. The proposed signal representation was evaluated using Monte Carlo simulations in identical parallel cylinders with hexagonal and random packing as well as parallel cylinders with gamma‐distributed radii. Results showed that the approach is sensitive to sizes in the interval 4–12 μm and exchange rates in the simulated range of 0 to 20 $s^{-1}$, but also that there is a sensitivity to the extracellular geometry. The presented theory constitutes a simple and intuitive description of how restricted diffusion and exchange influence the signal as well as a guide to protocol design capable of separating the two effects.

## Graphical abstract

Diffusion MRI acquisition protocols can be tuned to selectively probe restricted diffusion and exchange, effectively separating these otherwise entangled sources of signal contrast.

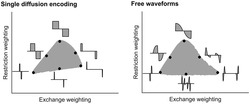

**Abbreviations used**
ADC: apparent diffusion coefficient
AXR: apparent exchange rate
CTI: correlation tensor imaging
DDE: double diffusion encoding
dMRI: diffusion MRI
FEXI: filter exchange imaging
FWF: free waveforms
IMPULSED: Imaging Microstructural Parameters Using Limited Spectrally Edited Diffusion
NEXI: neurite exchange imaging
OGSE: oscillating gradient spin echo
PGSE: pulsed gradient spin echo
POMACE: pulsed and oscillating gradient MRI for assessment of cell size and extracellular space
SDE: single diffusion encoding
SMEX: standard model with exchange
VERDICT: vascular, extracellular, and restricted diffusion for cytometry in tumors

## 1 INTRODUCTION

Diffusion MRI (dMRI) is an important radiological tool because it sensitizes the MR signal to the diffusion of water molecules, which indirectly probes tissue microstructure.^1^, ^2^, ^3^, ^4^, ^5^, ^6^ Today, dMRI provides clinically useful information about the microstructure via the apparent diffusion coefficient (ADC).^7^, ^8^, ^9^ Although the ADC is sensitive to pathology, it is not specific. This can be remedied by probing additional information pertaining to, for example, the time dependence of the diffusion process.^10^, ^11^ In particular, sensitizing the dMRI experiment to restricted diffusion and exchange enables inference of metrics sensitive to cell sizes and membrane permeability, respectively. However, restricted diffusion and exchange have opposite effects on the diffusion‐weighted signal^12^, ^13^, ^14^; as the diffusion time increases, the signal for a given encoding strength (b‐value) is elevated by restricted diffusion and reduced by exchange. Therefore, the estimation of, for example, cell sizes, can be confounded by a high membrane permeability, and vice versa. Current approaches often assume that restricted diffusion and exchange dominate in different time regimes; the former at short time scales and the latter at long time scales.^11^ This assumption enables straightforward estimation of one of the parameters while neglecting the other. Some approaches avoid that time‐scale assumption and incorporate both restricted diffusion and exchange,^11^, ^14^, ^15^, ^16^ using the standard pulsed gradient spin echo (PGSE) or the oscillating gradient spin echo (OGSE) sequences for the diffusion encoding. However, our preliminary work^13^ indicated that more efficient measurements of restricted diffusion and exchange can be achieved with gradient waveforms that can be arbitrarily modulated. This work explores when and how such benefits manifest.

Cell sizes can be estimated with dMRI in numerous ways. Early approaches used the diffraction‐like signal patterns in the narrow pulse limit to estimate restriction lengths in materials such as polystyrene spheres^17^ and erythrocytes.^18^ However, in vivo applications are challenging because exceptionally strong gradients are required to probe small structures,^19^, ^20^ and these systems are rarely used in human imaging.^21^ Distributions of cell sizes can also obscure diffraction patterns.^22^, ^23^, ^24^, ^25^ More recent approaches address this by instead utilizing compartment models that describe cells as impermeable objects in an extracellular medium.^11^, ^26^, ^27^ Examples applied for cancer imaging include Imaging Microstructural Parameters Using Limited Spectrally Edited Diffusion (IMPULSED),^28^ pulsed and oscillating gradient MRI for assessment of cell size and extracellular space (POMACE),^29^, ^30^ and vascular, extracellular, and restricted diffusion for cytometry in tumors (VERDICT).^31^ IMPULSED quantifies compartment sizes and the intracellular fraction using both OGSE and PGSE acquisitions. POMACE also exploits both PGSE and OGSE measurements in different time scales to estimate the extracellular volume fraction, cell size, and compartment diffusivities. VERDICT also uses PGSE, but incorporates a vascular compartment modeled by an anisotropic diffusion tensor. Sizes can also be estimated without using explicit tissue models. Nilsson et al.^32^ proposed an approach in which a Taylor series approximation of the diffusion spectrum is used to capture restriction‐driven time‐dependent diffusion. The approach was sensitive to restrictions larger than 4 μm using gradient strengths available at clinical MRI systems. Notably, the approach is also distinguished by its ability to accommodate diffusion encoding by arbitrary gradient waveforms.

Membrane permeability (reflected by the water exchange rate) has also been incorporated in diffusion analysis in multiple ways. Latour et al.^33^ proposed an effective medium theory describing the effect of permeability on diffusion in the long‐time regime. The approach was applied to estimate membrane permeability in bovine red blood cells^33^ and yeast.^34^ Novikov et al.^15^ treated cell membranes as randomly oriented flat layers to define a forward model that incorporated effects of permeability. This model was recently applied to estimate cell permeability and size in vivo.^20^ There are also restriction‐exchange approaches that are based on the standard model, which refers to a class of models comprising an intra‐axonal stick compartment and an isotropic Gaussian extra‐axonal component.^35^ Examples include SMEX (standard model with exchange)^36^ and NEXI (neurite exchange imaging),^37^ which both employ the Kärger model^38^ to describe exchange between the intra‐ and extra‐neurite spaces. All of these approaches employ a single pair of pulsed gradients for the diffusion encoding. However, using two pairs of pulsed gradients with a variable mixing time—referred to as double diffusion encoding (DDE)^39^—can be beneficial for exchange mapping.^40^, ^41^ Lasič et al.^42^ proposed filter exchange imaging (FEXI), which is sensitive to cell permeability captured by the so‐called apparent exchange rate (AXR). This parameter can differentiate between viable and necrotic parts of a tumor^43^ and has been applied to characterize breast tumors.^44^ Exchange‐related approaches were exclusively based on pulsed gradients until Ning et al.^45^ derived an approach that accommodates arbitrary gradient waveforms. The potential utility of such waveforms for exchange mapping has, however, not yet been systematically explored.

Sensitivity to tissue microstructure is governed by the experimental approach, here defined as the set of gradient waveforms used to acquire the dMRI data. As indicated earlier, several waveform designs have been proposed and applied in combination with mapping of restricted diffusion and exchange. The most commonly used waveform—here referred to as single diffusion encoding (SDE)^39^—comprises a pair of identical pulsed gradients inserted in a spin‐echo experiment.^46^ Although this approach is the main workhorse of dMRI, more complex waveforms may have benefits. For example, DDE—which uses two pairs of pulsed gradients^47^—can be used to map exchange^17^, ^48^ or restricted diffusion,^49^ whereas oscillating gradient waveforms have been suggested for probing diffusion at shorter time scales.^50^, ^51^, ^52^, ^53^ Both DDE and OGSE are fully specified by a few parameters such as pulse duration, pulse separation, and oscillation frequency. While this enables closed‐form expressions for the signal, it forgoes more intricate waveform designs that can increase efficiency or the amount of information encoded into the signal.^54^ Diffusion encoding by free waveforms (FWF) was first explored by Callaghan,^55^ who adopted the multiple propagator method^56^ and proposed a forward model for encoding restricted diffusion with arbitrary waveforms. Later, Drobnjak et al.^57^ optimized waveforms for maximized sensitivity to restricted diffusion. Waveforms have also been optimized for efficiency, tensor‐valued encoding, and suppression of various artefacts and motion encoding.^54^ Although waveform designs exist to emphasize the effects of either restricted diffusion or exchange separately, a unified framework that captures both phenomena independently using conventional and arbitrary gradient waveforms has not yet been proposed.

In this work we propose a signal representation that features effects of both restricted diffusion and water exchange. We arrive at this representation using a second‐order cumulant expansion of the signal attenuation due to diffusion‐induced phase dispersion. We highlight that the effect of the waveform on the second and fourth cumulants of the phase distribution can be evaluated in terms of a scalar quantity that indicates how much a given gradient waveform encodes for restricted diffusion.^32^ Similarly, we note that the fourth cumulant features a scalar quantity that describes how strongly a gradient waveform encodes water exchange.^45^ This approach unifies two separate frameworks from previous studies.^32^, ^45^ We employ the waveform‐dependent scalar parameters to characterize the restriction‐ and exchange‐weighting properties of different types of waveforms. Based on these metrics, we design a protocol that maximizes the statistical leverage in estimates of restriction length and exchange rate. We also investigate the value of free waveforms compared with designs based on SDE, DDE, and OGSE. Using Monte Carlo simulations on various substrates, we also identify the limits of applicability of the proposed representation. Finally, we apply the approach to analyze previously proposed experimental techniques to evaluate the degree to which they encode for restricted diffusion and exchange.

## 2 THEORY

In the following, the first section outlines the proposed restriction‐exchange representation. For simplicity, we consider here only the one‐dimensional case (i.e., diffusion encoding in one direction at a time). The representation is based on several approximations and expresses the relationship between features of the diffusion process on the one hand and features of the encoding gradient waveform on the other. The second section provides an analysis of the expected values of the representation parameters in theoretically simple cases where the ground truth is known, which provides understanding and illustrates potential limitations associated with the approximations.

### 2.1 Deriving the restriction‐exchange signal representation

The attenuation of the MR signal, *S*, due to diffusion can be expressed in terms of the spin phase distribution and its cumulant expansion up to fourth order,^58^

(1) $$\ln\left(S/S_{0}\right)=\ln\left\langle \exp\left(-\mathrm{i\phi}\right)\right\rangle \;\approx \;-\frac{1}{2}{\left\langle \phi^{2}\right\rangle }_{c}+\frac{1}{24}{\left\langle \phi^{4}\right\rangle }_{c}=-\frac{1}{2}c_{2}+\frac{1}{24}c_{4},$$

where $S_{0}$ is the nondiffusion‐weighted signal, $\left\langle \cdot \right\rangle$ is an average over all spin trajectories contributing to the signal, and $c_{2}={\left\langle \phi^{2}\right\rangle }_{c}$ and $c_{4}={\left\langle \phi^{4}\right\rangle }_{c}$, where the subscript “c” denotes the cumulant. The phase ϕ is defined as the inner product of the gradient waveform, $g\left(t\right)$, and the spin trajectory, $r\left(t\right)$,

(2) $$\phi =\gamma \int_{0}^{T}g\left(t\right)\cdot r\left(t\right)dt,$$

where γ is the gyromagnetic ratio and T is the total encoding time, here defined as the total duration of $g\left(t\right)$. We begin by considering the second cumulant in a population of spins undergoing diffusion with similar characteristics (an environment), and express it as

(3) $$\frac{1}{2}c_{2}=\frac{1}{2\pi }\int_{-\infty }^{\infty }D\left(\omega \right)\cdot {\left|q\left(\omega \right)\right|}^{2}\mathrm{d\omega},$$

where $D\left(\omega \right)$ is the diffusion spectrum and $\;{\left|q\left(\omega \right)\right|}^{2}$ is the diffusion encoding power spectrum given by ${\left|q\left(\omega \right)\right|}^{2}={\left|\text{FT}\left(q\left(t\right)\right)\right|}^{2}$, where FT denotes Fourier transform and $q\left(t\right)$ is the dephasing q‐vector related to the gradient waveform via $q\left(t\right)=\gamma \int_{0}^{t}g\left(\tau \right)d\tau .$ ^59^ We proceed with the following representation of the diffusion spectrum

(4) $$D\left(\omega \right)\;\approx \;\beta_{0}+\beta_{2}\omega^{2},$$

where $\beta_{0}$ and $\beta_{2}$ are constants that represent the time‐independent and time‐dependent parts of $D\left(\omega \right)$. It should be noted that the expression in Equation (4) does not describe the diffusion process in disordered media, where $D\left(\omega \right)\propto \omega^{p}$ and where *p* can assume values different from 0 and 2.^35^, ^60^, ^61^ This puts a limitation on the applicability of the framework and later sections will discuss the extent of this limitation in more detail. The approximation in Equation (4) allows the second cumulant to be written as

(5) $$\frac{1}{2}c_{2}\;\approx \;\frac{1}{2\pi }\int_{-\infty }^{\infty }\left(\beta_{0}+\beta_{2}\omega^{2}\right)\cdot {\left|q\left(\omega \right)\right|}^{2}d\omega =b\beta_{0}+{\mathrm{bV}}_{\omega }\beta_{2}.$$

Note that this representation features two diffusion‐related parameters ( $\beta_{0}$ and $\beta_{2}$) and two waveform‐related parameters (*b* and $V_{\omega }$). The first waveform parameter is the conventional b‐value, defined as $b=\int_{0}^{T}q{\left(t\right)}^{2}\mathrm{dt}$, and the latter captures the variance of the encoding spectrum, and is defined as^32^

(6) $$V_{\omega }=\frac{1}{2\mathrm{\pi b}}\int_{-\infty }^{\infty }{\left|q\left(\omega \right)\right|}^{2}\omega^{2}\mathrm{d\omega}=\frac{\gamma^{2}}{b}\int_{0}^{T}g^{2}\left(t\right)dt.$$

This parameter controls the restriction weighting of the waveform. In a voxel with multiple environments, the global second cumulant (denoted $c_{2}$) becomes the average of the second cumulants within each environment ( $o_{2}$) such that

(7) $$\frac{1}{2}c_{2}=\frac{1}{2}{\left\langle o_{2}\right\rangle }_{e}\;\approx \;bE_{\beta_{0}}+{bV}_{\omega }E_{\beta_{2}},$$

where ${\left\langle \cdot \right\rangle }_{e}$ denotes averaging across multiple environments and thus $E_{\beta_{i}}={\left\langle \beta_{i}\right\rangle }_{e}$. The fourth cumulant, in the absence of exchange, becomes^62^

(8) $$c_{4}={\left\langle o_{4}\right\rangle }_{e}+3\left({\left\langle o_{2}^{2}\right\rangle }_{e}-{\left\langle o_{2}\right\rangle }_{e}^{2}\right)\;\approx \;12b^{2}\cdot \left[V_{\beta_{0}}+2V_{\omega }C_{\beta_{0}\beta_{2}}+V_{\omega }^{2}V_{\beta_{2}}\right],$$

where the first term ${\left\langle o_{4}\right\rangle }_{e}$ represents the average fourth cumulant that is intrinsic to each environment. Here we approximate it to be zero, but we note that other studies suggest otherwise.^63^, ^64^ The second term represents the variance across environments, where

(9) $$V_{\beta_{i}}={\left\langle \beta_{i}^{2}\right\rangle }_{e}-{\left\langle \beta_{i}\right\rangle }_{e}^{2}$$

is the variance in $\beta_{i}$ ( i=0,2) and

(10) $$C_{\beta_{0}\beta_{2}}=\left({\left\langle \beta_{0}\beta_{2}\right\rangle }_{e}-{\left\langle \beta_{0}\right\rangle }_{e}{\left\langle \beta_{2}\right\rangle }_{e}\right)$$

is the correlation between $\beta_{0}$ and $\beta_{2}$.

Exchange between environments manifests as a decrease in the observed variance. We postulate that this can be represented in the form of a function $h\left(k\right)$ as follows

(11) $$c_{4}\;\approx \;12b^{2}\cdot \left[V_{\beta_{0}}+2V_{\omega }C_{\beta_{0}\beta_{2}}+V_{\omega }^{2}V_{\beta_{2}}\right]\cdot h\left(k\right),$$

where k is the exchange rate. Note that, in general, the three components of the variance may have different dependencies on the exchange rate, but here we assume $h\left(k\right)$ to be a universal description of the effect of exchange on all sources of variance. In our representation, we adopt the functional form of $h\left(k\right)$ from previous work on exchange in Gaussian environments,^45^ yielding

(12) $$h\left(k\right)=2\int_{0}^{T}\tilde{q_{4}}\left(t\right)\exp\left(-\mathrm{kt}\right)dt,$$

with $\tilde{q_{4}}\left(t\right)=q_{4}\left(t\right)/b^{2}$, where $q_{4}\left(t\right)$ is the fourth‐order autocorrelation function of the dephasing q‐vector given by $q_{4}\left(t\right)=\int_{0}^{T}q^{2}\left(t^{\prime }\right)q^{2}\left(t^{\prime }+t\right)dt^{\prime }$. We refer to $q_{4}\left(t\right)$ as the exchange‐weighting function. Note that Equation (12) is straightforward to extend to situations with multiple exchange rates, but that is beyond the scope of the present analysis. Assuming the product kt is small enough to allow the approximation $\exp\left(-\mathrm{kt}\right)\;\approx \;1–\mathrm{kt}$, the function $h\left(k\right)$ can be written as

(13) $$h\left(k\right)\;\approx \;1-k\Gamma ,$$

where

(14) $$\Gamma =2\int_{0}^{T}t\;\tilde{q_{4}}\left(t\right)dt.$$

This parameter controls the exchange weighting of the waveform. By combining the effects of restriction and exchange, we arrive at a unified signal representation, according to

(15) $$\ln\left(S/S_{0}\right)\;\approx \;-b\cdot \left[E_{\beta_{0}}+V_{\omega }E_{\beta_{2}}\right]+\frac{1}{2}b^{2}\cdot \left[V_{\beta_{0}}+2V_{\omega }C_{\beta_{0}\beta_{2}}+V_{\omega }^{2}V_{\beta_{2}}\right]\cdot \left(1-k\Gamma \right).$$

The value of this representation is that it generalizes to any gradient waveform by capturing its effect on the signal via three parameters ( b, $V_{\omega }$, and Γ). Furthermore, it highlights that, under the previously considered approximations, the time dependence of the diffusion process manifests in six parameters ( $E_{\beta_{0}}$, $E_{\beta_{2}}$, $V_{\beta_{0}}$, $C_{\beta_{0}\beta_{2}}$, $V_{\beta_{2}}$, and k).

### 2.2 Significance of the representation in various environments

The representation parameters are by nature sensitive to different microstructural features. Here, we clarify this by deriving expected values of the representation parameters in theoretically simple environments. This also allows us to illustrate limitations in interpretation. The analysis considers increasingly complex situations, starting with a single pool of freely diffusing spins and ending with a mixture of Gaussian and restricted environments. This allows us to illustrate where and how limitations in interpretation arise.

#### 2.2.1 One Gaussian pool

Free diffusion results in a Gaussian phase distribution.^59^ The diffusivity is captured in the first term $\beta_{0}=D_{0}$, where $D_{0}$ is the bulk diffusion coefficient. The absence of time dependence gives $\beta_{2}=0$. Because of the lack of diffusional variance, all cumulants of order greater than 2 are zero, meaning that $V_{\beta_{0}}=C_{\beta_{0}\beta_{2}}=V_{\beta_{2}}=0.$ We expect an application of the representation to this scenario to provide correct estimates of $D_{0}$, except for measurement errors caused by, for example, the rectified noise floor.^65^, ^66^, ^67^

#### 2.2.2 Two nonexchanging Gaussian pools

Consider two nonexchanging unrestricted pools associated with fractions $f_{1}$ and $f_{2}$ and diffusivities $D_{1}$ and $D_{2}$. Similar to the single Gaussian pool case, we have $\beta_{0}=D_{1}$ and $\beta_{2}=0$ for the first pool and $\beta_{0}=D_{2}$ and $\beta_{2}=0$ for the second. The average diffusivity is given by the weighted average of compartment diffusivities^45^, ^68^

(16) $$E_{\beta_{0}}=f_{1}D_{1}+f_{2}D_{2}.$$

The only source of variance is the difference in diffusivities between the two pools. Lack of time dependence yields $C_{\beta_{0}\beta_{2}}=V_{\beta_{2}}=0$ and thus^45^, ^68^

(17) $$V_{\beta_{0}}=f_{1}{\left(D_{1}-E_{\beta_{0}}\right)}^{2}+f_{2}{\left(D_{2}-E_{\beta_{0}}\right)}^{2}=f_{1}f_{2}{\left(D_{1}-D_{2}\right)}^{2}.$$

As the representation is based on a truncated cumulant expansion, we expect estimates of $E_{\beta_{0}}$ and $V_{\beta_{0}}$ to be biased because of the influence of higher‐order terms.^62^ This bias is expected to increase with the maximal b‐value.^62^, ^69^

#### 2.2.3 Two exchanging Gaussian pools

Water exchange between two pools that exhibit Gaussian diffusion has been explored using the Kärger model.^38^ An analysis of this type of situation shows exchange has no effect on the mean diffusivity.^45^ However, the variance is reduced according to

(18) $$V\;\approx \;V_{\beta_{0}}\cdot \left(1-k\Gamma \right),$$

where $k=k_{12}+k_{21}$ and $k_{\mathrm{ij}}$ is the exchange rate from pool i to j. Because the exchange‐weighting parameter Γ was defined using a first‐order approximation (Equation 13), Equation (18) is valid only when the product *k* Γ is small, that is, for relatively slow exchange or weak exchange‐weighting. Consequently, we expect an underestimation of *k* for situations where the product *k* Γ is large.

#### 2.2.4 One restricted compartment

Restricted diffusion in regular geometries (cylinders, spheres, or parallel planes) can be described in the frequency domain.^59^ At low frequencies, $D\left(\omega \right)$ can be approximated by its Taylor series expansion

(19) $$D\left(\omega \right)\;\approx \;R\omega^{2},$$

where R is what we refer to as the restriction coefficient, which for the regular geometries has the general form^32^, ^59^, ^70^

(20) $$R=cd^{4}D_{0}^{-1},$$

where d represents the compartment size, $D_{0}$ is the intrinsic diffusivity, and c is a constant with a value of 7/1536 for a cylinder and 1/500 for a sphere.

Consider a case where the MR signal emanates from a single restricted compartment. We expect all terms of our representation related to Gaussian diffusion to be zero, that is, $E_{\beta_{0}}=0$ and $V_{\beta_{0}}=C_{\beta_{0}\beta_{2}}=0.$ By matching the coefficients from the approximation above to those in the representation, we expect that

(21) $$E_{\beta_{2}}=R.$$

Note that while $E_{\beta_{2}}$ is constant, the apparent diffusion coefficient is time dependent and is given by $\mathrm{ADC}=V_{\omega }E_{\beta_{2}}$. This illustrates the role of $V_{\omega }$ as the parameter controlling the sensitivity of the experiment to restricted diffusion. The parameter $V_{\omega }$ was defined by approximating the diffusion spectrum with a second‐order Taylor series expansion (Equation 4). This approximation diverges from the true diffusion spectrum at higher frequencies. If $E_{\beta_{2}}$ is estimated using a protocol featuring waveforms with considerable power at such high frequencies, we expect a negative bias in $E_{\beta_{2}}$ as the quadratic approximation exaggerates $D\left(\omega \right)$ at higher frequencies. The bias is expected to increase with the underlying restriction length (size), as the frequency at which the approximation fails decreases with size.^32^ We also note that $V_{\omega }$ is not valid for irregular geometries such as undulating fibers, which can exhibit nonquadratic diffusion spectra at time scales relevant for MRI.^61^

The variance term that is related to restricted diffusion is the within‐compartment cumulant,^63^ ${\left\langle o_{4}\right\rangle }_{e}$, presented in Equation 8) and is expected to be negative.^71^, ^72^ However, none of the parameters of our representation captures this effect, and we therefore expect a bias in size estimates when the b‐value is high enough to render the variance term relevant. An overestimation of the variance is expected to lead to an underestimation of size.

#### 2.2.5 One hindered compartment

The diffusion spectrum in environments where diffusion is hindered has been shown to follow a power law scaling in the limit ω→0, ^35^, ^60^, ^70^, ^73^ such that

(22) $$D\left(\omega \right)\;\approx \;D_{\infty }+C\cdot R{\left(i\omega \right)}^{\vartheta },$$

where C is a constant, R denotes real part, and the dynamical exponent ϑ indicates the degree of disorder in the environment. Assuming two dimensions and short‐range disorder (which is the most common class),^35^ ϑ=1 and the diffusion spectrum takes the form

(23) $$D\left(\omega \right)\;\approx \;D_{\infty }+C\cdot \left|\omega \right|.$$

In this case, we expect $E_{\beta_{0}}$ to be sensitive mainly to $D_{\infty }$ and $E_{\beta_{2}}$ to C, as the latter is the parameter sensitive to time‐dependent diffusion. However, the representation fails to account for the linear dependence in ω because of how we approximated the diffusion spectrum ( $D\left(\omega \right)\;\approx \;\beta_{0}+\beta_{2}\omega^{2}$). Therefore, we expect a protocol‐dependent “crosstalk” between the two parameters where, depending on the waveform, a change in *C* may result in a change in $E_{\beta_{0}}$. The extent to which this happens will be analyzed using simulations. As later sections will highlight, a possible remedy to this challenge is the use of high b‐values that suppress the signal contribution from hindered compartments such as the extracellular space.

#### 2.2.6 Mixture of nonexchanging Gaussian and restricted compartments

Consider two nonexchanging compartments exhibiting Gaussian and restricted diffusion that have signal fractions $f_{G}$ and $f_{R}$. The time‐independent second‐order parameter, $\beta_{0}$, is now expected to represent the average of time‐independent diffusivities across the voxel as given by $E_{\beta_{0}}=f_{G}{\left\langle D_{0}\right\rangle }_{G}+f_{R}{\left\langle 0\right\rangle }_{R}=f_{G}{\left\langle D_{0}\right\rangle }_{G}$. Subscripts G and R represent averaging over the Gaussian and restricted environments, respectively. The averaging brackets here account for the possibility of a voxel containing multiple values of $D_{0}$ and R. The parameter $\beta_{2}$ is now expected to capture time dependence driven by the average restriction coefficient across compartments, yielding $E_{\beta_{2}}=f_{G}{\left\langle 0\right\rangle }_{G}+f_{R}{\left\langle R\right\rangle }_{R}=f_{R}{\left\langle R\right\rangle }_{R}.$ The total second‐order term becomes

(24) $$E=E_{\beta_{0}}+V_{\omega }E_{\beta_{2}}.$$

In this scenario, we expect $E_{\beta_{0}}$ to correctly capture the second‐order time‐independent characteristics of the system and $E_{\beta_{2}}$ to capture restriction‐driven time dependence at low frequencies and small sizes. The variance term features contributions from all three compartments according to

(25) $$V=\mathrm{Var}\left(\beta_{0}+V_{\omega }\beta_{2}\right)=V_{\beta_{0}}+V_{\omega }^{2}V_{\beta_{2}}+2V_{\omega }C_{\beta_{0}\beta_{2}},$$

where $V_{\beta_{0}}$ is the variance in time‐independent diffusivities across compartments and is given by

(26) $$V_{\beta_{0}}={\left\langle \beta_{0}^{2}\right\rangle }_{G,R}-{{\left\langle \beta_{0}\right\rangle }_{G,R}}^{2}=f_{G}{\left\langle {\left(D_{0}-E_{\beta_{0}}\right)}^{2}\right\rangle }_{G}+f_{R}{\left\langle {\left(0-E_{\beta_{0}}\right)}^{2}\right\rangle }_{R},$$

$V_{\beta_{2}}$ represents the variance in restriction coefficients across compartments

(27) $$V_{\beta_{2}}={\left\langle {\beta_{2}}^{2}\right\rangle }_{G,R}-{{\left\langle \beta_{2}\right\rangle }_{G,R}}^{2}=f_{G}{\left\langle {\left(0-E_{\beta_{2}}\right)}^{2}\right\rangle }_{G}+f_{R}{\left\langle {\left(R-E_{\beta_{2}}\right)}^{2}\right\rangle }_{R},$$

and $C_{\beta_{0}\beta_{2}}$ is the covariance between $\beta_{0}$ and $\beta_{2}$ given by

(28) $$C_{\beta_{0}\beta_{2}}={\left\langle \beta_{0}\beta_{2}\right\rangle }_{G,R}-{\left\langle \beta_{0}\right\rangle }_{G,R}{\left\langle \beta_{2}\right\rangle }_{G,R}.$$

We note that the same shortcomings as described in the previous sections apply here as well, but that the introduction of a mixture is not in itself a limitation. However, note that $E_{\beta_{2}}$ features a product of the restricted fraction and the average restriction coefficient that reflects the compartment sizes. This means that a low b‐value protocol probing only the second cumulant cannot disentangle fraction and size. Such disentanglement requires information from the fourth cumulant.

#### 2.2.7 Mixture of exchanging Gaussian and restricted compartments

The second cumulant is unaffected by exchange, provided the exchange is barrier limited.^45^, ^68^ Under this condition, we expect exchange effects to be well‐approximated by the Gaussian case, wherein

(29) $$V=\left[V_{\beta_{0}}+V_{\omega }C_{\beta_{0}\beta_{2}}+V_{\omega }^{2}V_{\beta_{2}}\right]\cdot \left(1-k\Gamma \right).$$

Note that an increasing exchange rate will challenge the assumptions underlying Equation (29). Indeed, when the product kΓ becomes large enough, the parameter Γ is no longer enough to describe the exchange‐weighting properties of the gradient waveform.^45^ The result is an underestimation of the underlying exchange rate. As the true exchange rate increases further, the second cumulant becomes dependent on exchange and the barrier‐limited assumption becomes void.^14^, ^74^ In that case, the entire framework presented in this work ceases to be applicable.

## 3 METHODS

### 3.1 Characterization of waveforms in the restriction‐exchange space

The theory highlighted two parameters ( $V_{\omega }$ and Γ) that describe how strongly a gradient waveform encodes for restricted diffusion and exchange, respectively. The first goal was to study these parameters for a large set of waveforms. Four waveform families were investigated: a single pair of pulsed gradients (SDE), two pairs of pulsed gradients (DDE), oscillating gradients in a spin echo (OGSE), and free waveforms (FWF). SDE waveforms were generated by varying the pulse width ( δ) and the pulse separation ( Δ), as described in Appendix S1. DDE waveforms were generated by varying the mixing time, the leading‐edge separation ( $\Delta_{1},\Delta_{2}$), and the pulse width ( δ), while assuming symmetric DDE, where $\delta_{1}=\delta_{2}$ and $\Delta_{1}=\Delta_{2}$. Pulse shapes were trapezoidal with the ramp time determined by the amplitude of the waveform and the maximum slew rate. OGSE waveforms included both sine‐ and cosine‐modulated varieties, and we varied the frequency of oscillation, the pulse duration ( δ), and the pause duration for an RF pulse ( Δ−δ). For FWF, a large set of waveforms aimed at spanning the set of executable gradients were generated by the procedure described in Appendix S1. All waveforms were subjected to the following constraints:

1. Maximum gradient amplitude of 80 mT/m at a b‐value of 0.5 ms/μm^2^
2. Maximum slew rate of 70 T/m/s
3. Minimum pause duration of 9 ms to allow for the refocusing pulse^75^
4. Total encoding time below 200 ms
5. Symmetry about the 180° refocusing pulse to satisfy the spin‐echo condition and avoid concomitant gradient effects.^76^

Waveforms generated for all four families (SDE, DDE, OGSE, and FWF) were mapped to the restricted‐exchange space, which was analyzed in terms of the size and shape of the region spanned by the waveforms.

We studied how well the generated waveforms complied with the assumptions made in the derivation of the restriction‐ and exchange‐weighting parameters $V_{\omega }$ and Γ. In the case of restriction, we characterized the error via a metric that captures the relative amount of encoding power at high frequencies:

(30) $$\eta_{d}=\frac{\int_{\omega_{\mathrm{thr}}\left(d\right)}^{\infty }{\left|q\left(\omega \right)\right|}^{2}d\omega }{\int_{0}^{\infty }{\left|q\left(\omega \right)\right|}^{2}d\omega },$$

where $\omega_{\mathrm{thr}}\left(d\right)$ is a threshold frequency, above which the $\omega^{2}$ approximation ceases to correctly represent the diffusion spectrum for a compartment of size *d*. The threshold frequency in the calculation of $\eta_{d}$ was defined as the frequency at which the relative difference between the full diffusion spectrum and the Taylor series expansion exceeded 10%. For exchange, we defined the metric $\eta_{k}$ that compares the full exchange‐weighting term $h\left(k\right)$ in Equation (12) and its approximation in Equation (13)

(31) $$\eta_{k}={\left(h\left(k\right)-\left(1-k\Gamma \right)\right)}^{2}.$$

For each waveform family, we identified the waveforms satisfying $\eta_{d}\leq 80\%,\eta_{d}\leq 40\%$, and $\eta_{d}\leq 20\%,$ as well as $\eta_{k}\leq 80\%,\eta_{k}\leq 40\%$, and $\eta_{k}\leq 20\%$. The metric $\eta_{d}$ was computed for a diameter of 20μm and $\eta_{k}$ for an exchange rate of 20 s^−1^. These values were chosen because they comprised the upper bound in the simulations (described later).

### 3.2 Investigating different protocols for fixed microstructure parameters

Having characterized waveforms in the restriction‐exchange space, we proceeded to evaluate the performance of the approach in Equation (15). For this purpose, a protocol comprising a set of waveforms and a set of b‐values was defined (details given below). Synthetic measurements were then performed using Monte Carlo simulations (described in Appendix S2). Model parameters were estimated from the simulated signals by fitting Equation (15) using the nonlinear least squares solver *lsqnonlin* in MATLAB (MathWorks, Natick, MA, USA; R2019a). A size index (*d*) was computed from the fitted parameter $E_{\beta_{2}}$ using $d={\left(D_{\text{in}}\cdot E_{\beta_{2}}/\left(c\cdot f_{\text{in}}\right)\right)}^{1/4}$. This computation featured the known underlying values of the intracellular diffusivity ( $D_{\text{in}}$) and signal fraction ( $f_{\text{in}}$) and was performed to simplify the presentation of the results. Note that this cannot be done when *D*_in_ and *f*_in_ are unknown, as they are in the general case.

The performance of the approach was evaluated by investigating the effect of the total encoding time $\left(T_{\max}\right)$ and maximum b‐value $\left(b_{\max}\right)$ on size and exchange estimates, knowing that increasing these tends to increase the violation of the assumptions underpinning the theory. To assess the performance of the approach, we studied how the bias and precision in parameter estimates as well as the effect size varied with $T_{\max}$ and $b_{\max}$. The parameter $T_{\max}$ was defined as the total duration of the longest‐lasting waveform in a given protocol. Bias was defined as the absolute difference between the parameter value expected from the simulations and the value obtained from the fitting. Precision was defined as the standard deviation across multiple fittings on data with uniquely generated noise. The effect size (Cohen's d) expected from a group study was calculated as

(32) $$ℇ=\frac{\bar{X_{1}}-\bar{X_{2}}}{S_{X_{1}X_{2}}},$$

where $\bar{X_{1}}-\bar{X_{2}}$ is the difference in estimated means between two simulated groups and $S_{X_{1}X_{2}}$ denotes the pooled standard deviation of the two groups.

To find the combination of $T_{\max}$ and $b_{\max}$ with the lowest bias, highest precision, and largest effect size, we investigated protocols defined with $T_{\max}$ varying from 50 to 200 ms and $b_{\max}$ ranging from 0.5 to 10 $\mathrm{ms}/\mu m^{2}$. Enforcing a given $T_{\max}$ and $b_{\max}$ reduced the set of candidate waveforms to a compact subregion in the restriction‐exchange space. We then chose four waveforms maximally separated on the convex hull of this region. The four waveforms were scaled to achieve b‐values in the interval 0 to $b_{\max}$. At each combination of $T_{\max}$ and $b_{\max}$, signals were simulated for cylinder diameters of 1 and 5 μm and exchange rates of 0 and 5 $s^{-1}$ using the substrate with randomly packed parallel cylinders. Effect size estimates were computed for these two settings using Equation (32). Rice‐distributed noise was added to the signals at SNR = $200\cdot \exp\left(-T_{\max}/T_{2}\right),$ where we assumed $T_{\max}=\mathrm{TE}$ and $T_{2}=80$ ms.

### 3.3 Investigating different microstructures for a fixed protocol

Our next aim was to analyze the performance of the approach under different microstructures but a fixed protocol. We expected this analysis to illustrate the conditions under which the representation succeeds and fails at providing parameters with a strong link to the ground truth.

For each waveform family, a fixed test protocol was defined using waveforms selected from the restriction‐exchange space after applying the constraints $T\leq T_{\mathrm{opt}}$ and $b_{\max}\geq b_{\mathrm{opt}}$, where $T_{\mathrm{opt}}$ and $b_{\mathrm{opt}}$ are the optimal maximum encoding time and b‐value that gave the best compromise between bias and precision in the preceding section. Four waveforms were selected such that they were maximally separated on the convex hull of the region of waveforms satisfying the named constraints. Where possible, the waveforms were chosen such that one pair would strongly encode restriction (maximum separation in $V_{\omega }$) while the other one strongly encoded exchange (maximum separation in Γ). The waveforms in each protocol were scaled in amplitude to attain the maximum b‐value $b_{\mathrm{opt}}$ found in the previous section.

The performance of the approach was evaluated by analyzing the bias, crosstalk, and precision. For this analysis, signals were simulated using the fixed protocols and all combinations of cylinder diameter and exchange rate from the sets $d=\left\{1,2,3,4,5,6,8,10,12,14,16,18,20\right\}\;\mathrm{\mu m}$ and $k=\left\{0,1,2,3,4,5,6,8,10,12,14,16,18,20\right\}\;s^{-1}$. All the other simulation parameters were kept fixed at $f_{\text{in}}=1-f_{\mathrm{ex}}=0.5,D_{\text{in}}=D_{\mathrm{ex}}=2\;\mu m^{2}/\mathrm{ms}$. The performance was evaluated using simulations in three substrate types of increasing complexity: hexagonally packed equally sized parallel cylinders, randomly packed parallel cylinders of equal size, and randomly packed parallel cylinders of Gamma‐distributed diameters. In the last case, the mean diameter and mean exchange rate were varied to match the values supplied above and the ground truth size was defined as ${\left(\left\langle d^{6}\right\rangle /\left\langle d^{2}\right\rangle \right)}^{1/4}$. Bias was defined as the absolute difference between the estimate and the ground truth value. Crosstalk was defined as the impact of one parameter on another, for example, a size‐dependent bias in the exchange rate. Precision was assessed by adding Rice‐distributed noise at a generous SNR of 200 (at b = 0) to signals simulated for randomly packed cylinders with the following combinations of parameters: (diameter, exchange rate) = (4 μm, 2 $s^{-1}$), (8 μm, 2 $s^{-1}$), and (8 μm, 10 $s^{-1}$). The fitting procedure was in all cases the same as the one described in ection 3.2.

### 3.4 Visualizing common protocols in the restriction‐exchange space

Finally, to understand how previously published protocols for probing of restricted diffusion and/or exchange manifest in the restriction‐exchange space, we computed and visualized Γ and $V_{\omega }$ for the waveforms used in SDE optimized for estimation of Kärger model parameters,^77^ FEXI,^42^ IMPULSED,^28^ OGSE,^78^ and correlation tensor imaging^63^ (CTI). Note that only the part of the CTI protocol featuring parallel gradient pairs is considered here. Table 1 summarizes the parameters of the five protocols.

**TABLE 1:** Gradient waveform parameters for five different protocols

Protocol	Parameters
SDE^77^	$\delta =\left[7,\;3.9,\;5.7,\;9.4\right]\;\mathrm{ms};$ $\;\Delta =\left[102,\;412,\;406,\;169\right]\;\mathrm{ms}$
FEXI^42^	$\delta_{f}=11\;\mathrm{ms};\delta_{d}=9\;\mathrm{ms};\;t_{f}=21\;\mathrm{ms};\;t_{d}=22\;\mathrm{ms};$ $\;t_{m}=\left[30,\;100,\;200,\;410\right]\;\mathrm{ms}$
OGSE^78^	$f=\left[20,\;40,\;60,\;200\right]\;\mathrm{Hz}$ $\text{pause duration}:\left(\Delta -\delta \right)=5\;\mathrm{ms};\;\delta =50\;\mathrm{ms}$
IMPULSED^28^	$\delta_{\mathrm{SDE}}=4\;\mathrm{ms};\;\Delta_{\mathrm{SDE}}=48\;\mathrm{ms}$ $\delta_{\text{OGSE}}=20\;\mathrm{ms};\Delta_{\text{OGSE}}=25\;\mathrm{ms};f_{\text{OGSE}}=\left[50,100,150\right]\;\mathrm{Hz}$
CTI^63^	$\delta =1.5\;\;\mathrm{ms};\;\Delta =13\;\mathrm{ms};\;t_{m}=13\;\mathrm{ms}$ $\left(\left|q_{1}\right|,\left|q_{2}\right|\right)=\left[\left(q_{b},q_{b}\right);\left(q_{b},q_{b}\right);\left(q_{b},0\right);\left(0,q_{b}\right)\right]$ $q_{b}=\sqrt{\left(b_{\max}/\left(\Delta -\delta /3\right)\right)}$

Abbreviations: CTI, correlation tensor imaging; FEXI, filter exchange imaging; IMPULSED, Imaging Microstructural Parameters Using Limited Spectrally Edited Diffusion; OGSE, oscillating gradient spin echo; SDE, single diffusion encoding; δ𝑓/δ𝑑, filter/detection block pulse width; $t_{f}/t_{d}$, filter/detection block diffusion time; $\;t_{m}$, mixing time.

## 4 RESULTS

### 4.1 Characterization of waveforms in the restriction‐exchange space

**FIGURE 1:**
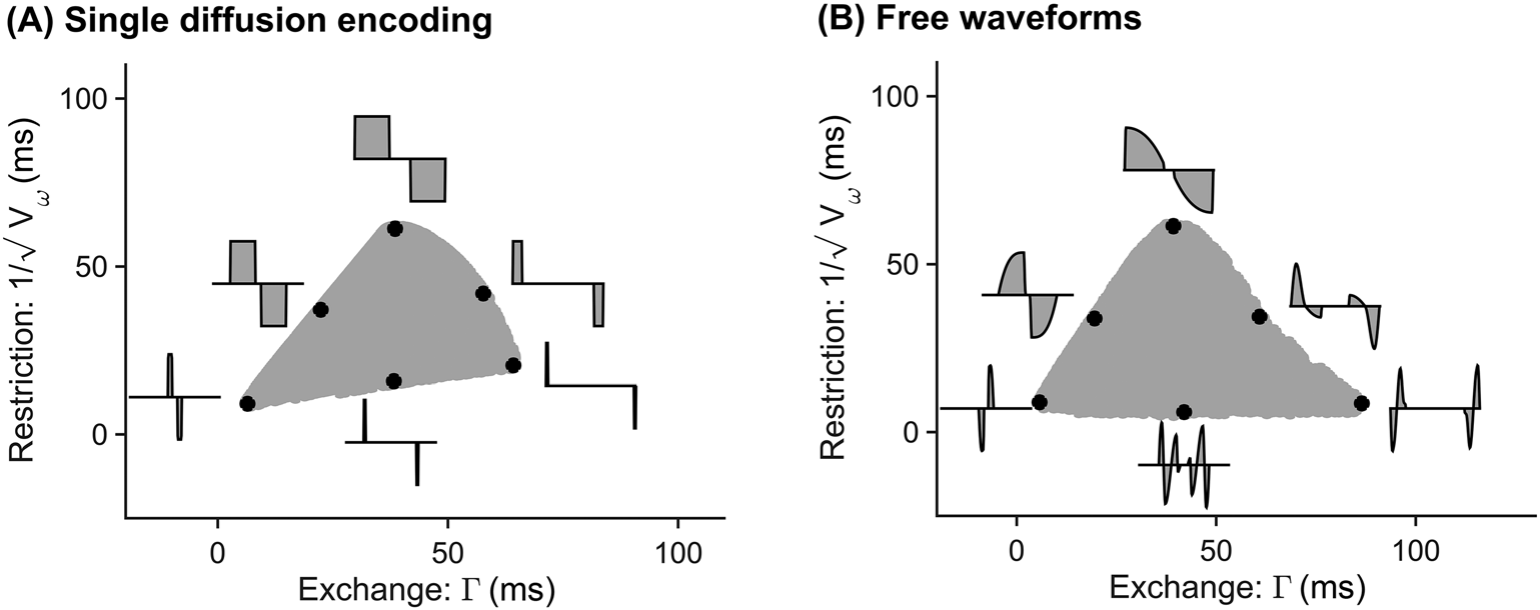
The restriction‐exchange space for all available waveforms from two families: (A) Single diffusion encoding (SDE) and (B) Free waveforms (FWF). The space is defined by Γ and $V_{\omega }$, which quantify the strength of exchange and restriction weighting for a given gradient waveform. Note that the y‐axis in this figure shows the inverse square root of $V_{\omega }$ to obtain the same unit and scale on both axes. Black points show the position of waveform examples, which were all plotted on the same time axis with a maximal duration of 200 ms. Waveforms were for both SDE and FWF generated with a maximum encoding time of 200 ms, a maximum gradient amplitude and slew rate of 80 mT/m and 70 T/m/s generating a minimum b‐value of 0. $5\;\mathrm{ms}/\mu m^{2}$. Note that the analysis shown here uses effective waveforms with an implied refocusing pulse in the middle

Our analysis of a large set of waveforms showed that these form a compact region in the restriction‐exchange space. Figure 1 visualizes the space in terms of $V_{\omega }^{-1/2}$ and Γ, which both have the unit of seconds. In the SDE case (panel A), narrow pulses gave the strongest restriction encoding (shortest $V_{\omega }^{-1/2}$). An increase in the diffusion time led to an elevated exchange encoding (longer Γ). Increasing both the pulse width and the diffusion time led to stronger exchange encoding but weaker restriction encoding. In the FWF case (panel B), different types of waveforms clustered into subregions populated by waveforms resembling SDE, DDE, or OGSE. The left edge was exclusively populated by SDE‐like waveforms. Such waveforms are the most efficient and high efficiency was needed in the left edge to deliver the required b‐value at low Γ, for which the total encoding time is short. SDE‐like waveforms also yielded the lowest $V_{\omega }$ because of their lack of oscillations. At longer Γ, DDE‐like waveforms were more common in the bottom of the region (high $V_{\omega }$) as DDE oscillates more than SDE. The region peaked at a Γ‐value of 40 ms because of the condition imposed on the maximum encoding time (200 ms). The right half of the triangular region comprised only oscillating waveforms because oscillations result in an exchange‐weighting function ( $q_{4}$) with nonzero values at longer times, which gives a longer Γ. Oscillations also add power at high frequencies of the encoding spectrum, which results in higher $V_{\omega }$. Taken together, these factors explain the increase in $V_{\omega }$ with increasing Γ to the right of the peak.

**FIGURE 2:**
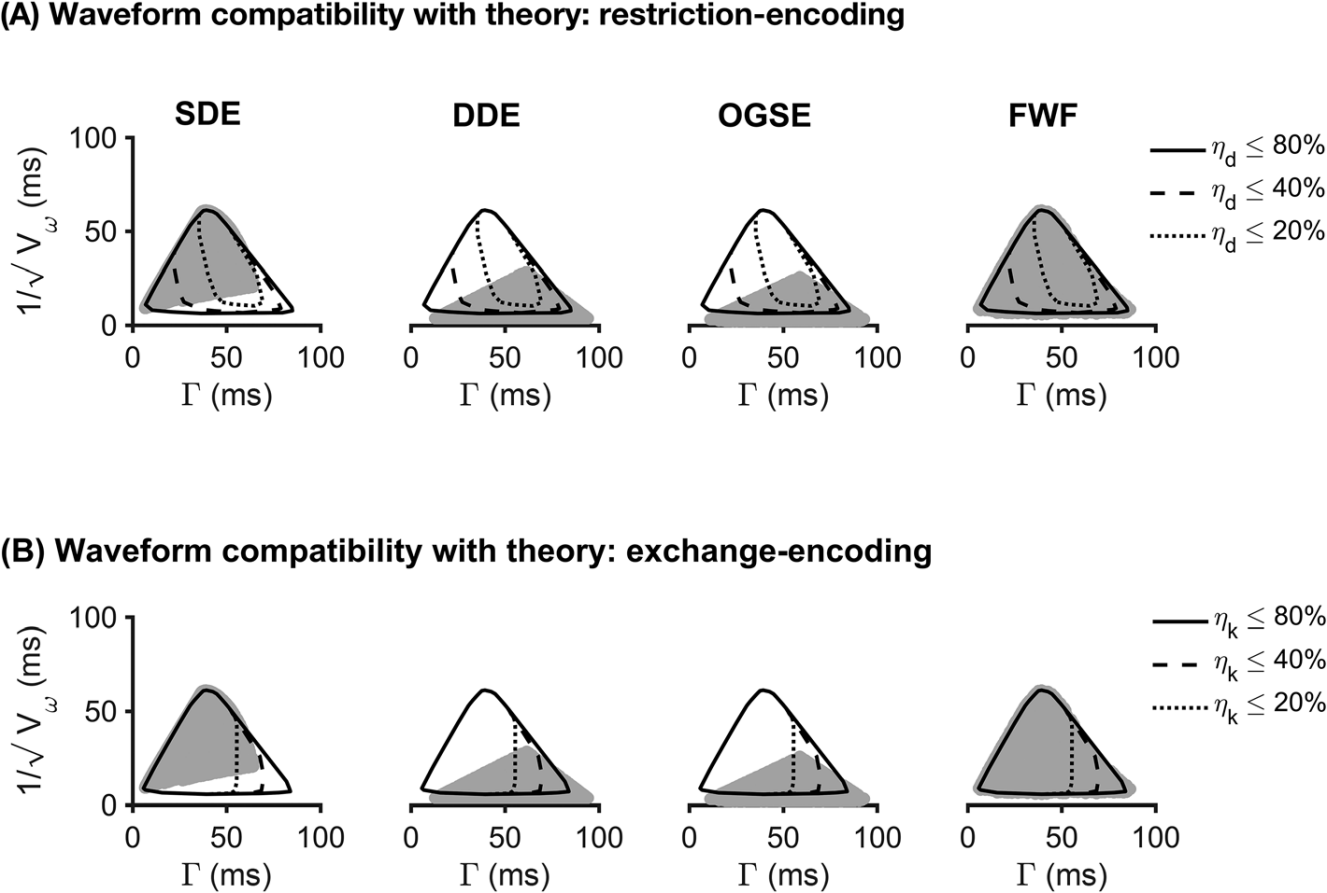
Waveform compatibility with assumptions for four waveform families: single diffusion encoding (SDE), double diffusion encoding (DDE), oscillating gradient spin echo (OGSE), and free waveforms (FWF). The shaded gray area represents the set of waveforms from each family that satisfy the basic constraints listed in section 3.1. (A) shows how well waveforms comply with the restriction‐related assumption as quantified by $\eta_{d}$ (Equation 30). As the requirement is sharpened (lower $\eta_{d})$, the number of waveforms complying with it gradually shrinks (solid, dashed, and dotted lines). (B) shows the corresponding analysis for the parameter $\eta_{k}$ (Equation 31), which quantifies how well waveforms comply with the exchange‐related assumption behind the theory. A sharper waveform requirement (smaller percentage) is associated with a smaller region

Figure 2 shows the restriction‐exchange space for four types of waveforms: SDE, DDE, OGSE, and FWF together with the outlines showing where waveforms comply with assumptions in terms of exhibiting low values of $\eta_{d}$ and $\eta_{k}$. Given any combination of constraints, FWF provides a wider range of candidate waveforms than SDE, which in turn provides a wider range than both DDE and OGSE. The reason the regions made up by DDE and OGSE candidates are notably smaller than those made up by SDE or FWF candidates is that the former cannot achieve high b‐values at short encoding times. Additionally, they can only provide strong exchange‐ and restriction‐weighting because of their oscillatory behavior. When considering the demands on $\eta_{d}$ and $\eta_{k}$, we can see that using stricter constraints on $\eta_{d}$ results in a near‐total exclusion of DDE and OGSE candidates. This occurs because such waveforms have substantial encoding power at higher frequencies. More stringent demands on $\eta_{k}$ exclude longer waveforms and constrains the set of candidates in a somewhat predictable manner by excluding the rightmost part of the waveform region.

### 4.2 Study of different protocols given fixed microstructure parameters

**FIGURE 3:**
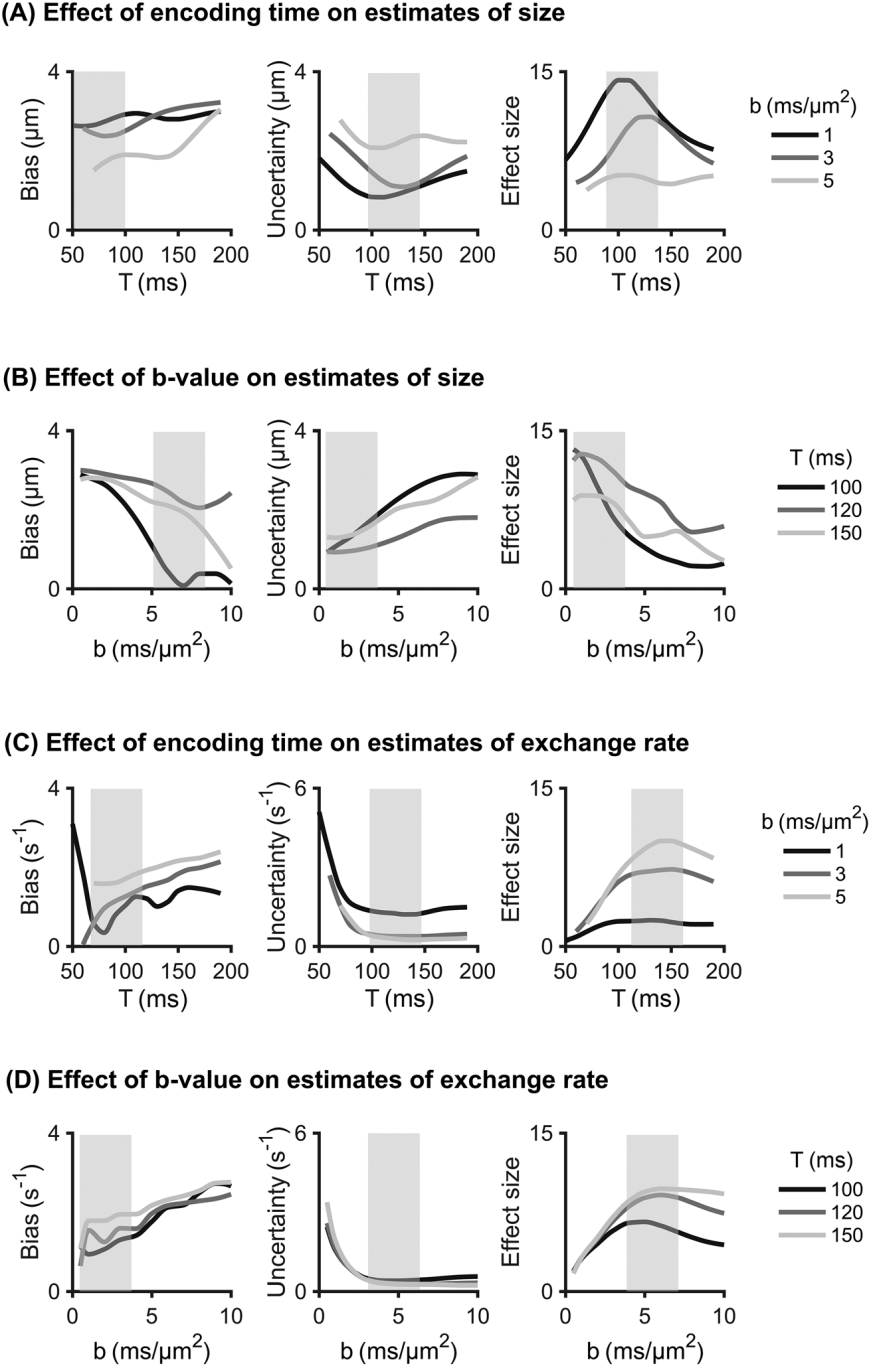
Bias, precision, and effect size in compartment size and exchange rate as a function of maximum encoding time ( $T_{\max}$) and maximum b‐value ( $b_{\max}$). Precision is quantified by the standard deviation (labeled “uncertainty” in the figure). SNR was set to 200 at TE = 0 and b = 0 and a T2 relaxation time of 80 ms was assumed. In (A), the favored range of values of $T_{\max}$ (least bias, highest precision, and largest effect size) is 100–150 ms. (B) shows that the favored $b_{\max}$ lies around 2 $\mathrm{ms}/\mu m^{2}$. Applying the same procedure to the exchange‐related results in (C) and (D), the desired values of $T_{\max}$ and $b_{\max}$ are around 100–150 ms and 5 $\mathrm{ms}/\mu m^{2}$. Collectively, this figure suggests an optimum where $T_{\max}$ is approximately 120 ms and $b_{\max}$ approximately 5 $\mathrm{ms}/\mu m^{2}$

To understand how the set of waveforms that make up a protocol influences performance in terms of parameter estimation, we evaluated the effect of the maximum encoding time $\left(T_{\max}\right)$ and maximum b‐value $\left(b_{\max}\right)$ on bias, precision, and effect size. Results are shown in Figure 3 for waveforms from the FWF family. Largely similar results were obtained using SDE waveforms (not shown). Two important concepts are illustrated in Figure 3. Increasing the maximum encoding time increases the range of Γ that a given protocol can achieve, and because of increased leverage, the precision in estimates of exchange rate is improved. However, increasing the maximum encoding time also prolongs the echo time, which in turn reduces SNR. Eventually the decrease in SNR overpowers the benefit of increased leverage and overall performance declines. A similar argument can be made regarding the maximum b‐value. In detail, panel A shows the size‐related results for protocols with variable $T_{\max}$. An interval is highlighted between 100 and 150 ms where the precision and effect size are at their maximum, but the bias is also high. Panel B shows size‐related results for variable $b_{\max}$. Maximum precision and effect size occur around a $b_{\max}$ of 2 $\mathrm{ms}/\mu m^{2}$, where bias is also high. Regarding exchange estimates, panel C shows a decline in bias up to some $T_{\max}$, above which the bias begins to grow. Both precision and effect size peak in the interval 100–150 ms. In panel D, the minimum bias occurs at low b‐values, but maximum precision and largest effect size occur around a $b_{\max}$ of 5 $\mathrm{ms}/\mu m^{2}$. Following these results, $T_{\max}=120$ ms and $b_{\max}=5$ $\mathrm{ms}/\mu m^{2}$ were selected as suitable compromises for the following analyses.

**FIGURE 4:**
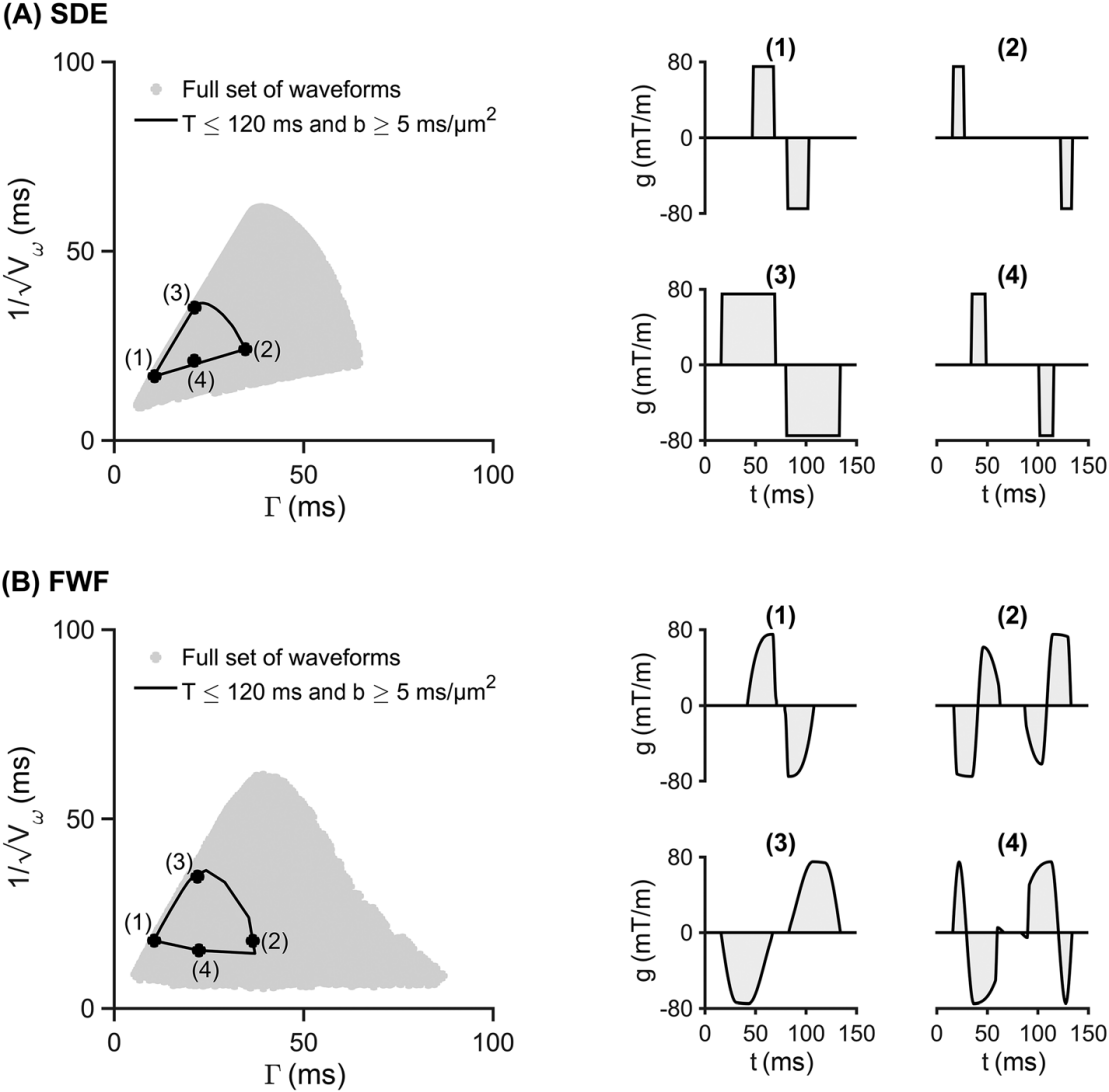
The final single diffusion encoding (SDE) and free waveforms (FWF) protocols comprising four waveforms. (A) shows the SDE restriction‐exchange space alongside the selected four‐waveform protocol and (B) shows the corresponding result for FWF. The four waveforms were selected among groups with a maximum encoding time of 120 ms, gradient amplitude and slew rate of 80 mT/m and 70 T/m/s, respectively, which were found within the region marked by a black line. The black dots mark the positions of the selected waveforms. For the SDE protocol, the ( Γ, $V_{\omega }^{-1/2}$) values for waveforms 1 to 4 are, in order, (11, 17), (35, 24), (21, 35), and (21, 21) ms. For FWF, these numbers are (11, 18), (37, 18), (22, 35), and (22, 15) ms. Thus, waveforms 1 and 2 have the same values of $V_{\omega }$ but different Γ and waveforms 3 and 4 have the same Γ but different $V_{\omega }$ *.* The range of Γ values between waveforms 1 and 3 is 26 ms for FWF and 24 ms for SDE

Figure 4 shows one FWF and one SDE protocol selected for further studies. Both protocols feature a total encoding time of 120 ms, a maximal b‐value of $5\;\mathrm{ms}/\mu m^{2}$ (very few waveforms fulfilled these criteria for DDE and OGSE), and four different waveforms. The FWF protocol comprised two waveforms with the same restriction‐weighting but different exchange‐weighting (waveform 1 and 2) and two waveforms with equal exchange‐weighting but different restriction‐weighting (3 and 4). The rationale behind this configuration is that the first pair of waveforms maximizes exchange‐driven contrast while the second pair maximizes restriction‐induced contrast. In the SDE case, this cross‐like configuration could not be achieved, so the first waveform pair (1 and 2) features both variable exchange and restriction weighting.

**FIGURE 5:**
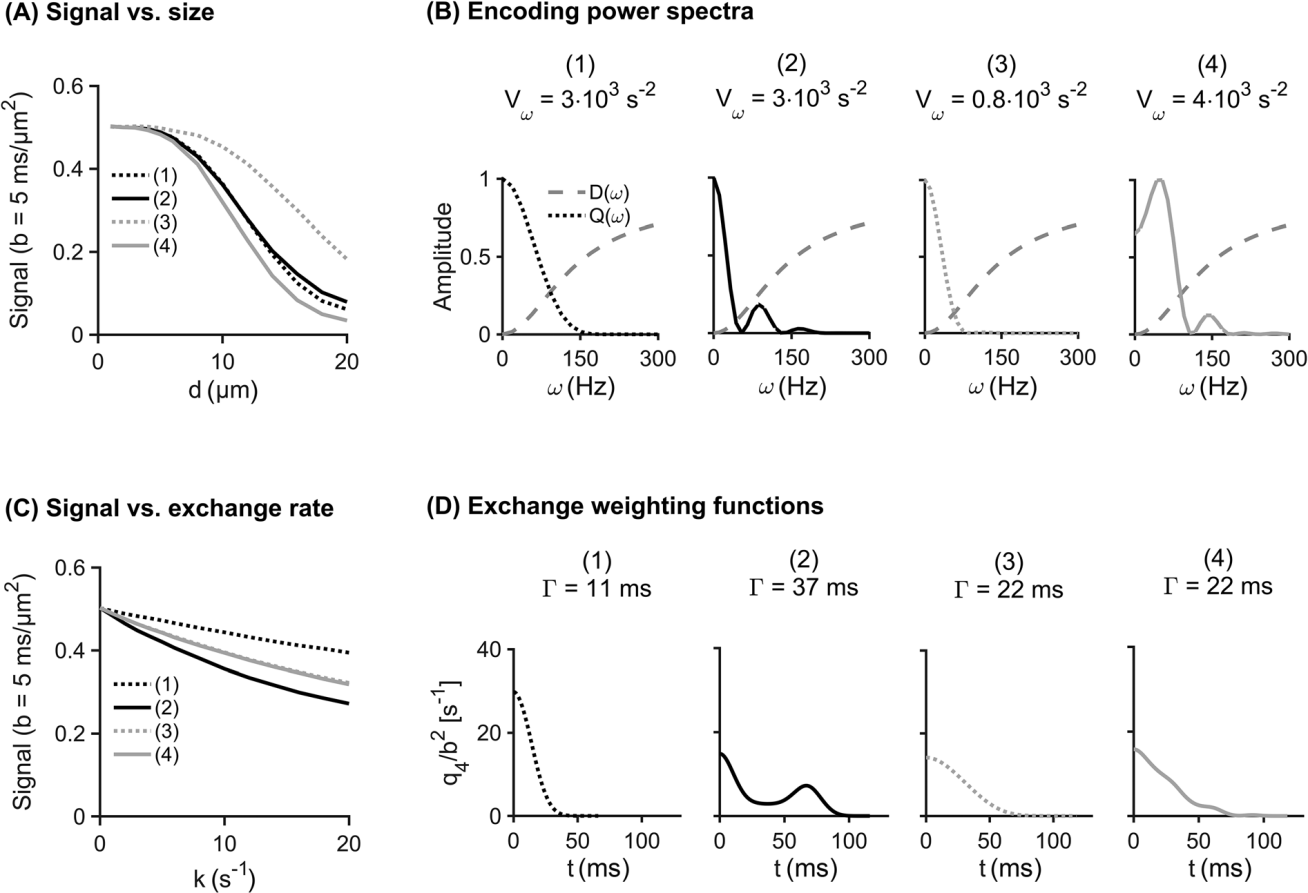
Illustration of the restriction and exchange encoding performed by the free waveforms in Figure 4. In (A), waveforms 3 and 4 have the weakest and strongest restriction weighting, respectively. This follows from that the power spectrum of waveform 4 contains power at higher frequencies than that of waveform 3 as shown in (B). waveforms 3 and 4 have equal exchange weightings and should therefore show no exchange‐driven contrast. Indeed, their signals in (C) coincide. In (C), waveforms 1 and 2 have the weakest and strongest exchange‐weighting, respectively. This is evident from the $q_{4}$ functions in (D), where waveform 2 features nonzero values at longer times than waveform 1. Their restriction weightings are equal, which means that they show no contrast with varying diameter. (A) shows that waveforms 1 and 2 begin to exhibit some contrast when the diameter grows above 12 μm. The signals shown were generated using a substrate with randomly packed identical cylinders

Figure 5 elucidates the mechanisms explored in this study using the FWF protocol. Panel A shows the signal at b = 5 $\mathrm{ms}/\mu m^{2}$ for cylinder diameters in the range 1 to 20 μm and panel B shows the encoding power spectrum for each waveform in the protocol alongside the diffusion spectrum for a cylinder with a diameter of 10 μm. Waveforms 1 and 2 have the same $V_{\omega }$ and—as predicted—yield the same signal for diameters below about 12 μm. For larger diameters, the approximations of the representation are no longer valid and the signals diverge for waveforms 1 and 2. As the restriction encoding is no longer described by $V_{\omega }$ alone, changes in diameter may erroneously be interpreted as changes in exchange rate. Panel C shows the signal at b = 5 $\mathrm{ms}/\mu m^{2}$ for microstructures defined by a diameter of 1 μm and exchange rates varying from 0 to 20 $s^{-1}$. Panel D shows the exchange‐weighting function ( $q_{4}$) for the four waveforms. As expected, waveforms 1 and 2 produce the maximal exchange‐driven signal contrast, while waveforms 3 and 4 have intermediate and overlapping signals. Contrary to the case for restricted diffusion, waveforms selected to exhibit equal exchange encoding yield the same signals regardless of the ground truth exchange rate.

### 4.3 Protocol performance for different microstructures

**FIGURE 6:**
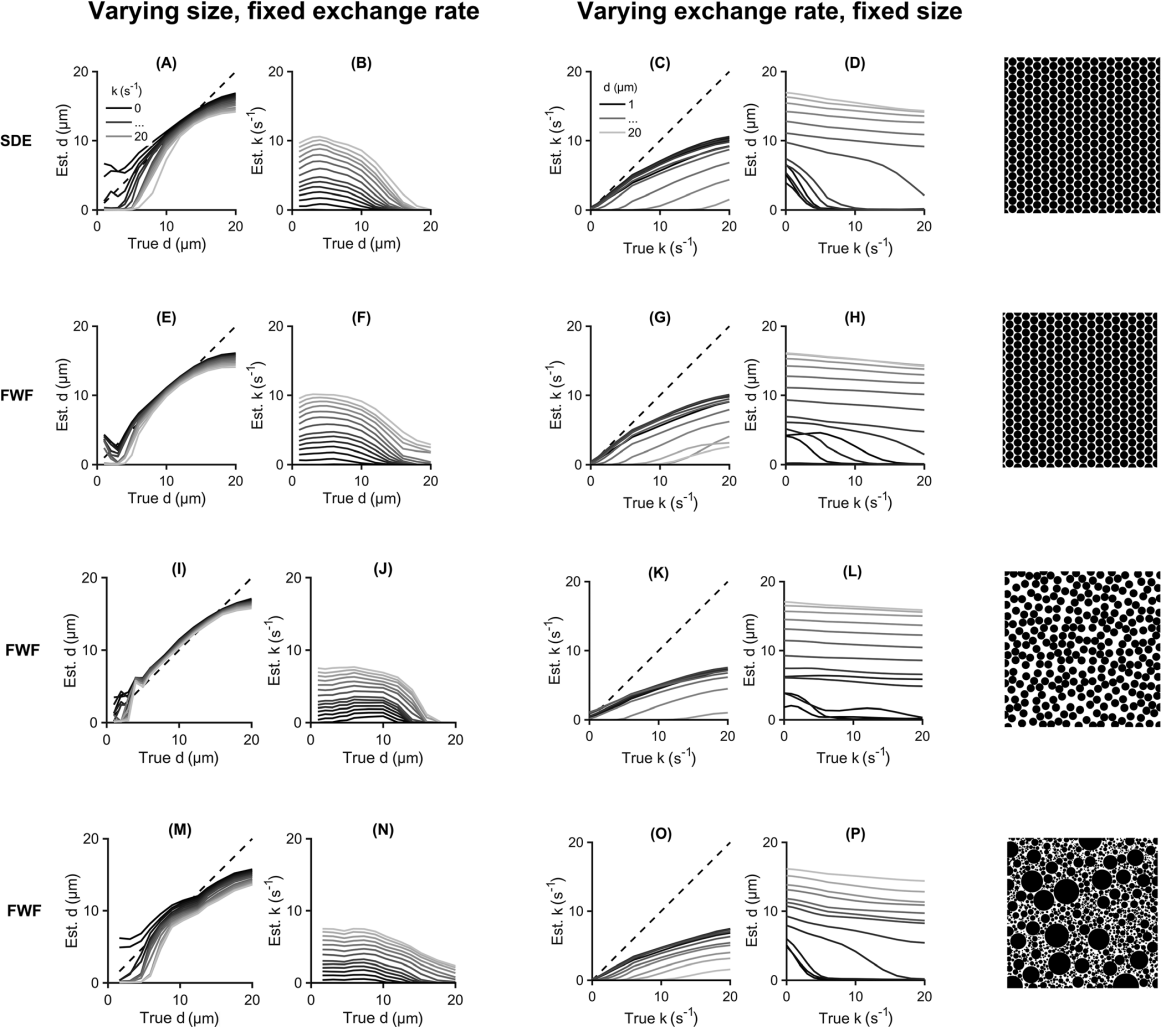
Bias and crosstalk in estimates of size and exchange rate in different diffusion environments (hexagonally packed, randomly packed, and gamma‐distributed cylinders). Simulations were performed using ground truth cylinder diameters and exchange rates from the sets 1, 2, 3, 4, 5, 6, 8, 10, 12, 14, 16, 18, and 20 μm and 0, 1, 2, 3, 4, 5, 6, 8, 10, 12, 14, 16, 18, and 20 $s^{-1}$, respectively. Sizes of 4 to 12 μm are well captured in substrates featuring a single size (A, E, I) with inferior estimates being obtained in the presence of a size distribution (M). Varying size has no effect on exchange estimates up to a size of 12 μm, after which there is some correlation between the two quantities (B, F, J, N). Free waveforms (FWF) show an advantage over single diffusion encoding (SDE) in this regard. Plots (C, G, K, O) show that the bias in exchange rate increases with exchange. Plots (D, H, L, P) present the crosstalk between size and exchange, where the size is faithfully captured at all exchange rates, except for the smallest sizes. There is a general decline in performance with increasing substrate complexity. Another general trend is that small sizes give higher crosstalk between size estimates and ground truth exchange rate, but lower crosstalk between exchange estimates and ground truth size. This is because the time dependence at small sizes is dominated by exchange, which benefits exchange estimation but makes size estimation a challenge. Overall, Figure 6 shows that the proposed representation is applicable for barrier‐limited exchange and sizes in the range 4–12 μm

Figure 6 shows the evaluation of bias and crosstalk for the SDE and FWF protocols. Results are shown for the three substrates considered: hexagonally packed cylinders (A–H), randomly packed cylinders (I–L), and gamma‐distributed cylinders (M–P). In the hexagonally packed substrate, the representation correctly reflected the underlying size in the interval 4 to 12 μm (E). Larger sizes challenge the approximations of the theory and smaller sizes deliver indistinguishable signal contrasts. Random packing led to a slight overestimation of sizes (panel I), which is in line with an extracellular time dependence (see section 2.2). For the gamma‐distributed cylinders there was a drop in sensitivity above an average size of 10 μm. Concerning crosstalk between varying diameter and estimated exchange rate, Figure 6 suggests that FWF outperforms SDE as it demonstrates less correlation between the two parameters over the range of simulated sizes (panels B and F). This result is likely a manifestation of the restriction‐exchange configurations presented in Figure 4, where the cross‐like configuration available for FWF has a noticeable advantage. Crosstalk in FWF estimates follows a similar pattern across all three types of substrates. Panels C and G show the bias in exchange estimates for variable exchange rates evaluated for the FWF and SDE protocols. The bias increased with increasing exchange rate. This result stems from two sources. The first is a violation of the time‐domain approximation made in the definition of the exchange‐weighting time. The second is the assumption of barrier‐limited exchange, which becomes increasingly void with increasing exchange rate. Crosstalk between size estimates and varying exchange rate was observed, in particular for smaller diameters. The level of crosstalk (slope of the curves) was larger for SDE than the FWF protocol (panels D and H). Across substrates, there was no clear difference in performance regarding crosstalk between size estimates and true exchange rate. Combinations of large sizes and fast exchange constitute a gross deviation from the approximations used to derive the representation, leading to the drastic decline in performance evident in panels C and G of Figure 6. Regarding variation across substrates, there is an overall decline in performance with increasing substrate complexity. Overall, Figure 6 suggests that the proposed representation is applicable to barrier‐limited exchange coupled with sizes in the range 4–12 μm. Note that the lower limit of this interval is imposed by hardware constraints rather than the theory.

**FIGURE 7:**
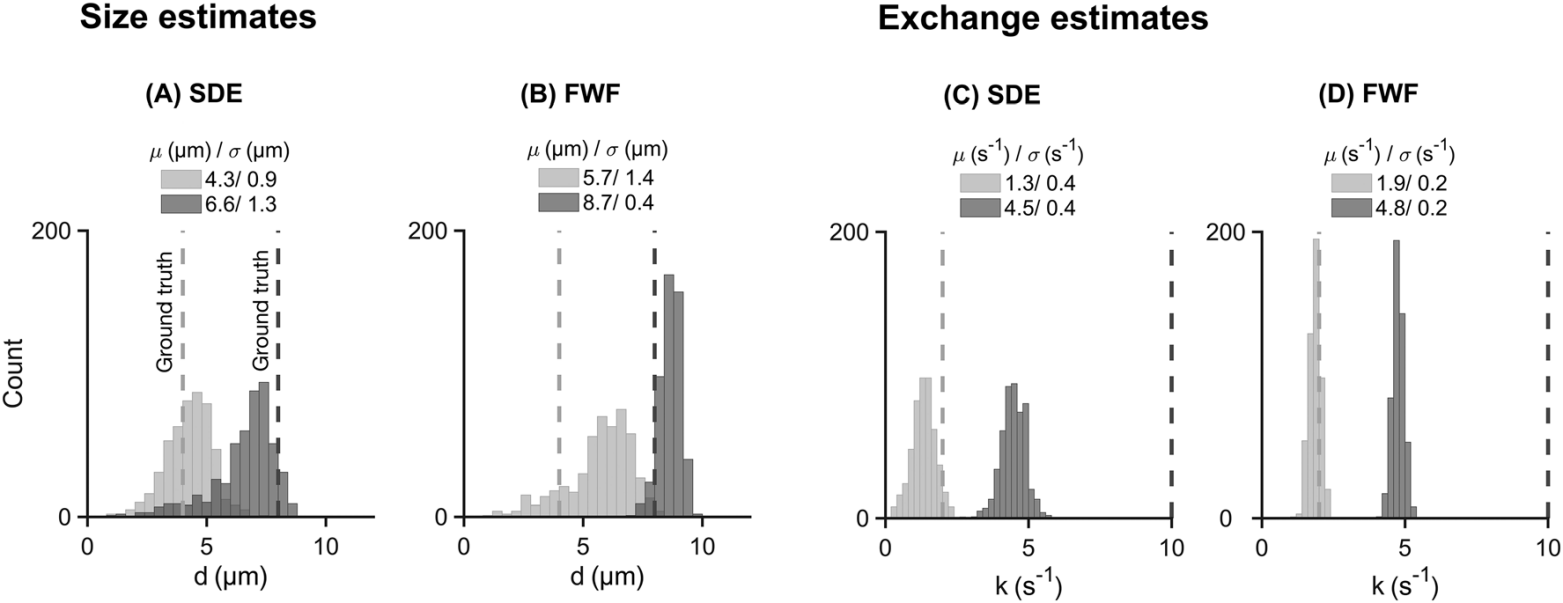
Estimates of compartment size and exchange rates: a comparison between single diffusion encoding (SDE) and free waveforms (FWF). Distributions of size estimates are shown for SDE in (A) and FWF in (B) while distributions of exchange estimates are shown in (C) and (D) for SDE and FWF, respectively. The ground truth sizes are 4 and 8 μm at a fixed exchange rate of 2 $s^{-1}$. For exchange, the ground truth values are 2 and 10 $s^{-1}$ at a fixed size of 8 μm. Simulated signals were generated using a substrate of identical randomly packed parallel cylinders. Symbols μ and σ represent the mean and standard deviation of estimates obtained at an SNR of 200 (at b = 0). For size estimates, FWF gives thrice the precision of SDE at the larger simulated diameter. There is a twofold improvement in precision in exchange estimates with FWF compared to SDE

Figure 7 shows a comparison between the SDE and FWF protocols in terms of precision. The parameter distributions follow the same patterns for both protocols. Apart from the case of a 4‐ μm cylinder diameter, precision in size estimates obtained with the FWF protocol is three times higher than that obtained with the SDE protocol (Figure 7). This result can be attributed to the larger range of restriction‐weightings ( $V_{\omega }$) delivered by the FWF protocol (Figure 4). This leverage is especially important at smaller sizes where restriction‐induced signal contrast is lower. Regarding exchange, Figure 7 shows that the FWF protocol adds a twofold improvement in precision compared with SDE, although estimates with both protocols are inaccurate. Taken together, the results of Figure 7 suggest that using FWF can improve the precision of parameter estimates by at least a factor of 2.

### 4.4 Visualizing common protocols in the restriction‐exchange space

**FIGURE 8:**
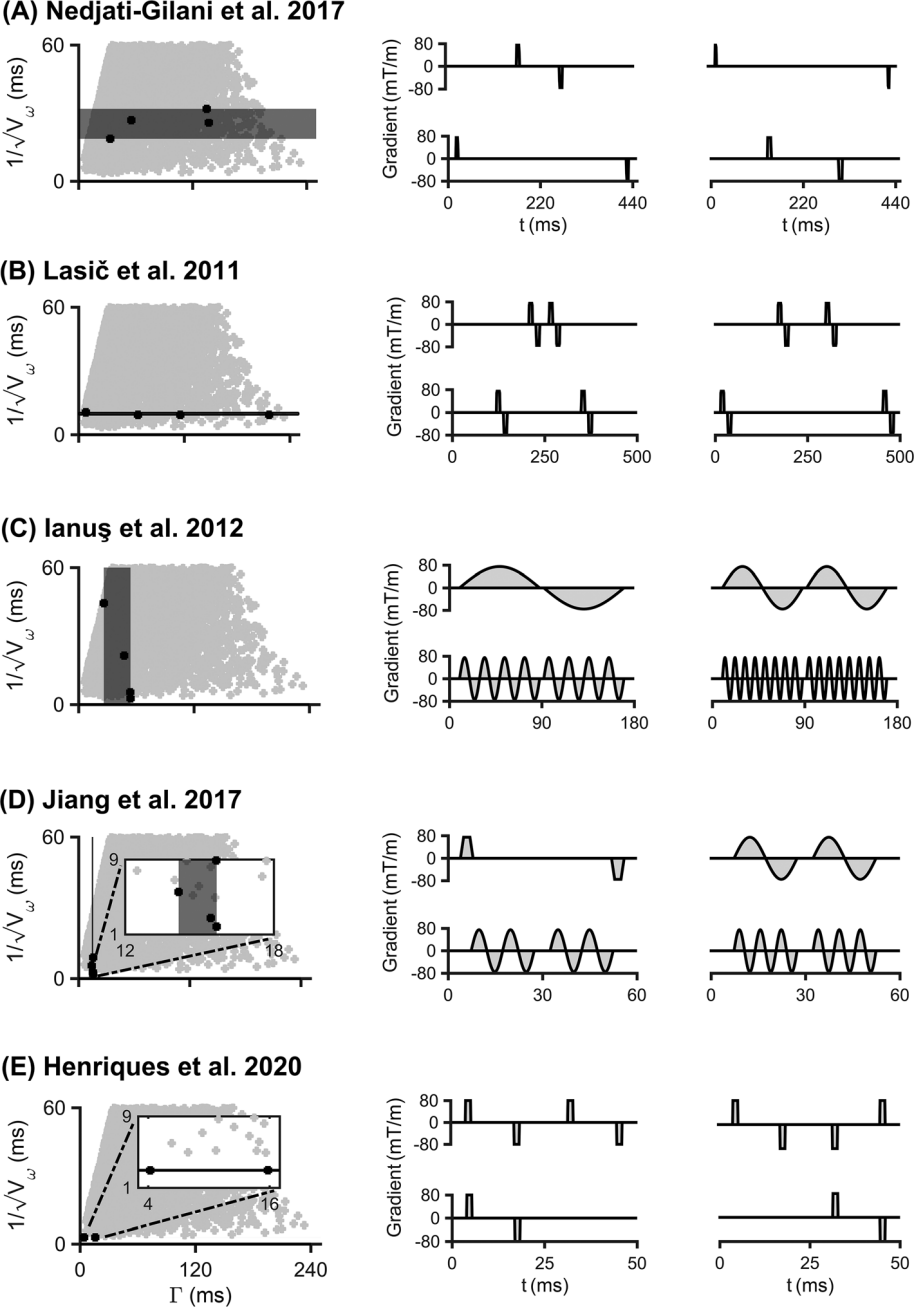
Visualization of five different protocols on the restriction‐exchange space: (A) single diffusion encoding (SDE) optimized for exchange estimation, (B) filter exchange imaging (FEXI), (C) oscillating gradient spin echo (OGSE), (D) Imaging Microstructural Parameters Using Limited Spectrally Edited Diffusion (IMPULSED), and (E) correlation tensor imaging (CTI). Note that only four out of eight waveforms from the CTI protocol are shown because the rest are a rescaling of those shown here, which does not change Γ and $V_{\omega }$. The restriction‐exchange space (gray region in the left column) was generated using free waveforms with a maximum gradient strength of 80 mT/m, slew rate of 70 T/m/s, and a maximum total encoding time of 500 ms. Each of the five protocols considered here comprise four waveforms shown adjacent to the restriction‐exchange space. The optimized SDE protocol is strongly sensitive to exchange but demonstrates greater restriction sensitivity than is seen in FEXI. FEXI lies along the exchange axis of the space, which means that the protocol is sensitive to exchange but not restriction. OGSE is sensitive to restriction but exhibits minor exchange sensitivity at low frequencies (< 50 Hz). IMPULSED is largely sensitive to restriction and shows negligible exchange sensitivity. CTI (assuming linear encoding) predominantly encodes exchange

Figure 8 provides a visualization of the restriction‐exchange weighting properties of waveforms from literature, including SDE optimized for exchange estimation, FEXI, OGSE, IMPULSED, and CTI. Here, the optimized SDE protocol is strongly sensitive to exchange but exhibits a greater restriction sensitivity than FEXI. FEXI demonstrates pronounced exchange sensitivity (variation in Γ) coupled with negligible restriction sensitivity (near constant $V_{\omega }$). OGSE is mostly sensitive to restriction, with a mild exchange sensitivity that tends to decrease with increasing frequency. IMPULSED strongly encodes restriction and is insensitive to exchange. CTI is sensitive to exchange (variable Γ), but not to restricted diffusion (constant $V_{\omega }$).

## 5 DISCUSSION AND CONCLUSION

In this work, we proposed and evaluated an approach for the encoding and analysis of restricted diffusion and water exchange in diffusion MRI. The approach features two aspects. The first is a signal representation that allows estimation of parameters connected to restricted diffusion and exchange. The second is the concept of mapping a gradient waveform to two scalar parameters that span the restriction‐exchange sensitivity space with one dimension for restricted diffusion^32^ and another for exchange.^45^ The utility and limitations of these aspects are discussed below.

Mapping waveforms into the restriction‐exchange sensitivity space provides useful insights. Here, we elaborate on three examples. First, we enable the selection of waveforms that maximize signal contrast due to either exchange or restriction, or both. For example, waveforms that lie on a horizontal line in the encoding space (varying Γ at constant $V_{\omega }$) provide variable sensitivity to exchange with constant sensitivity to restriction. Such waveforms enable accurate estimation of exchange without bias from restricted diffusion, assuming that the approximations given in Equation (4) (for restriction) and Equation (13) (for exchange) are valid. Second, we show that the set of available free waveforms span a triangular region in the restriction‐exchange space, with groups of similar waveforms clustered in distinct regions (Figure 1). Third, the restriction‐exchange sensitivity space can be used to analyze and compare previous approaches. In this work, we considered five examples from literature and found that the methods are variably specific to their stated purpose. For example, DDE (used in FEXI) and an optimized SDE protocol^77^ are both sensitive to exchange but not restriction, which is in line with the intended application of both protocols. Both OGSE and OGSE combined with SDE (as used in IMPULSED) predominantly encode restriction; however, changing the OGSE frequency also changes the exchange sensitivity. This may bias the results of studies that use OGSE for mapping restricted diffusion under the assumption of negligible exchange encoding.^79^, ^80^, ^81^, ^82^ The fifth and last example considered is CTI, which was recently proposed to disentangle restriction‐driven intracompartmental kurtosis $\left(\mathrm{\mu K}\right)$ from other kurtosis sources.^63^ Interestingly, our analysis suggests that CTI has sensitivity to exchange, which may confound the estimation and interpretation of μK. Alternatively, one could argue that exchange is to be regarded as one source of μK.

The numerical evaluation highlighted intervals of applicability of the proposed signal representation. For exchange, estimates showed a general bias, but this bias did not depend on the size until the point where the exchange was no longer barrier limited.^43^ For sizes larger than approximately 12 μm the bias was dependent on the size. Below this size, however, estimated exchange rates were linearly sensitive to true exchange rates of up to 20 $s^{-1}$. This applicability interval covers a subset of the conditions observed in vivo. For instance, previous work has reported exchange rates in the human brain in the range 1−40 $s^{-1}$.^43^, ^83^, ^84^, ^85^, ^86^, ^87^ Note that the exchange captured by the sensitivity refers to diffusional exchange in general, which encompasses but is not exclusive to permeation exchange across the cell membrane. Transition between anisotropic domains of different orientations would also be observed as exchange with the present approach.

In the case of restricted diffusion, the interval of applicability, where the estimates showed a linear sensitivity to the true parameters, covered sizes in the range of approximately 4 to 12 μm. For smaller sizes, the underlying compartment diameter approaches the resolution limit and thus cannot be mapped without bias.^32^, ^88^ Below this limit our size estimates also become dependent on the exchange rate. Accurate mapping of axonal sizes in the cerebrum is thus challenging with this approach, as these have diameter distributions with medians around 1 μm.^89^, ^90^ However, other approaches using ultrastrong gradients and custom experiments show promising results.^91^, ^92^ For sizes above 12 μm, the restriction‐weighting properties of a gradient waveform are no longer explained by $V_{\omega }$ alone (Figure 5). This scenario corresponds to the localization regime discussed in previous work.^32^, ^93^, ^94^, ^95^, ^96^ Note that this implies that waveforms that lie along the exchange axis of the restriction‐exchange space (such as FEXI) may become sensitive to restricted diffusion in environments with large structures. Indeed, previous work has shown that restricted diffusion can bias exchange rate estimates,^97^ which we refer to here as crosstalk (Figure 6). It is also worth noting that the bias in estimates observed with the current approach can in part be attributed nonzero intracompartmental kurtosis, which challenges the assumptions of the signal representation.^63^, ^64^, ^71^, ^72^ For diameters above the 12 μm threshold, microstructure models such as IMPULSED have been shown to yield more accurate estimates.^28^ However, while such microstructure models may provide better size estimates than the representation presented here, their accuracy hinges on the validity of the model. Regardless, any approach seeking to map only restricted diffusion must employ waveforms that provide equal sensitivity to exchange.

The use of either a signal representation or a microstructure model, with the terms used as defined in Novikov et al.,^98^ has implications on protocol design that warrant further discussion. Here we use the term representation for our signal model, as we make no explicit assumptions about the composition of the microstructure, but merely approximations regarding the characteristics of the diffusion process. As illustrated by Figure 2, the approximations used to derive our signal representation limit the set of waveforms that are compatible with the approach. While using incompatible waveforms translates to bias (Figure 6), restricting protocol design to a small set of waveforms reduces leverage, which translates to low precision. Furthermore, the performance analysis presented in Figure 3 showed a high optimal b‐value of 5 $\mathrm{ms}/\mu m^{2}$ coupled with a relatively short maximum encoding time of 120 ms, which excluded oscillating gradient waveforms as a feasible design. Thus, the approach is unable to leverage the strong restriction encoding delivered by such waveforms. Trade‐offs like this, where accuracy comes at the cost of precision, are commonplace in other cumulant‐expansion–based frameworks such as diffusion tensor imaging and diffusion kurtosis imaging.^69^, ^99^, ^100^ Using a model rather than a representation could allow a larger set of waveforms to be used, at the cost of parameter accuracy if assumptions are invalid.^101^ It is worth noting that, under all constraints placed on the restriction‐exchange space, free waveforms exhibited a larger coverage of the space compared with other waveform designs: an expected result due to their flexible shape.^13^, ^54^, ^76^ The benefit of using these waveforms was also highlighted by at least a twofold improvement in precision (halving of the standard deviation) compared with SDE. We expect that this advantage of FWF can be leveraged by a microstructure model that is compatible with all waveforms, but the development of such a model remains the target of future work.

We observed a decline in performance with increasing substrate complexity. The reason is that the representation in Equation (14) employs an $\omega^{2}$ approximation of the diffusion spectrum, which is invalid in disordered media. Such media have been shown to feature nonanalytic diffusion spectra.^35^, ^60^, ^70^, ^73^ In the presence of disorder, we expect an overestimation of the underlying restriction length, because $\beta_{2}$ becomes a conflation of effects from restriction and disorder. Regarding exchange, the decline in performance can be attributed in part to failure of the $\omega^{2}$ approximation and in part to failure of Equation (11), which assumed that all sources of variance are modulated by a common exchange‐dependent function $h\left(k\right)$. The effects of disorder can be alleviated by using strong diffusion weighting, which suppresses the extracellular signal contribution. We remark that this explains the tendency towards higher b‐values in the analysis shown in Figure 3. This technique is analogous to the application of strong diffusion weighting to suppress extracellular water and highlight the stick power‐law in white matter.^36^

Note that the effects of anisotropy were not covered by the current work. This shortcoming is important to address because diffusion shows considerable directional dependence in biological tissue. Microscopic diffusion anisotropy can be incorporated into a framework considering general waveforms and tensor‐valued diffusion encoding.^102^, ^103^, ^104^ A full multidimensional diffusion‐encoding framework developed by Lundell et al.^105^ and, more recently, Lasič et al.,^106^ can further disambiguate the effects of microscopic anisotropy and time‐dependent diffusion (excluding exchange). Previous work has shown that although the encoding of Gaussian diffusion may be isotropic, the exchange‐weighting parameter Γ varies across different gradient directions.^54^ A more complete forward model would account for anisotropic media and such a framework already exists for nonexchanging restrictions with the potential for extension to include exchange.^107^ Incorporating these findings and refining the theory accordingly is reserved for future work.

In conclusion, we have proposed a signal representation that offers an intuitive and explicit description of how the phenomena of restricted diffusion and water exchange manifest. Importantly, it provides insights into the restriction‐ and exchange‐weighting properties of gradient waveforms with arbitrary shapes. The approach provides an important guide in designing an experiment to maximize and disentangle the two independent contrasts.

## Supporting information


**Appendix S1:** Gradient waveform generation
**Appendix S2:** Monte Carlo simulations
