# Supplementary material for: Diffusion MRI with pulsed and free gradient waveforms: Effects of restricted diffusion and exchange
Source: NMR Biomed. 2022 Sep 27;36(1):e4827. doi: 10.1002/nbm.4827 (PMC10078514; doi:10.1002/nbm.4827)
Supplement: Supplementary file 1 — Appendix S1: Gradient waveform generation Appendix S2: Monte Carlo simulations [file NBM-36-0-s001.docx]

**Appendix A: Gradient waveform generation**

1. **Free waveforms (FWF)**


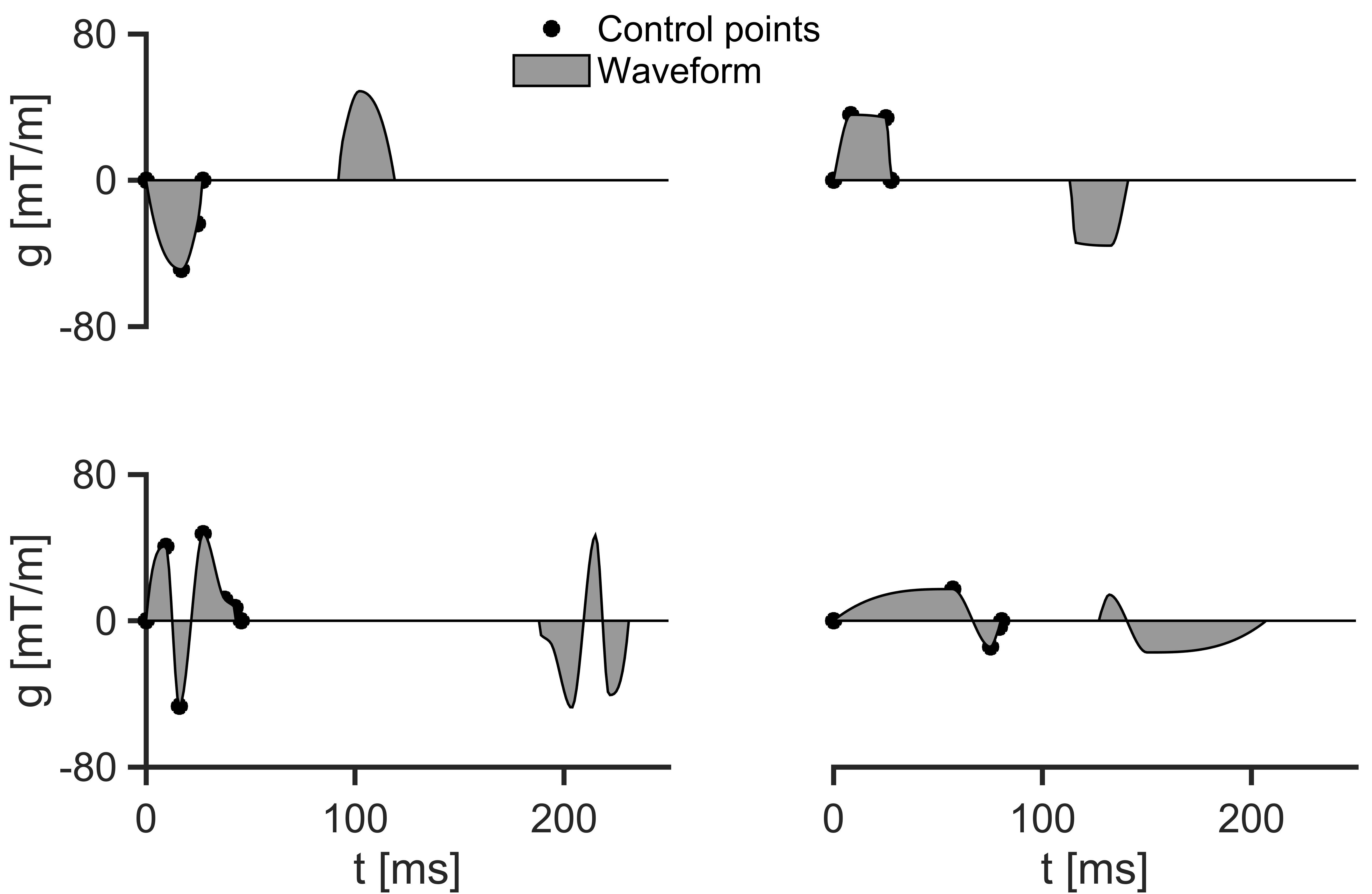


Figure A1. Generation of arbitrary gradient waveforms using control point interpolation

We began by generating a random set of control points according to

$$P_{i}=\left( x_{i},y_{i} \right)$$

where $i=1,2,\ldots,n$ and $n$ is the total number of control points. Note that, since the waveforms were designed to be mirror-symmetric, these control points were intended to produce the first gradient block (that is, the waveform in [0 T/2] where T is the total encoding time). The rest of the waveform would be a copy of this first block flipped in time. We generated control points satisfying the following constraints:

1. $\left( x_{1},y_{1} \right)=\left( 0,0 \right)$ and $y_{n}=0$ to ensure that the start and end values of the waveform were zero and that the waveform started at time zero.
2. $-g_{max}<y_{i}<g_{max}$ where $g_{max} = 80 mT/m$ is the maximum gradient amplitude. Note that the full waveform was then scaled to attain a b-value of 0.5 $\mathrm{ms}/\mu m^{2}$ and if, after this scaling, the waveform exceeded $g_{max}$, it would be thrown out.
3. $x_{i}\leq x_{i+1}$ to ensure an injective waveform (that is, one gradient value at each time point).
4. $x_{n}\leq\frac{1}{2}\left( T-t_{pause} \right)$ where $t_{pause}=9$ ms is the minimum pause time (the time between the end of the first and start of the second gradient block). This constraint ensures that the first gradient block does not use more than half of the maximum available encoding time and also that there is at least a time $t_{pause}$ between the two blocks. Note that in the case $x_{n}=t_{end}$ where $t_{end}<\frac{1}{2}\left( T-t_{pause} \right)$, the time interval $\left( t_{end},\frac{1}{2}\left( T-t_{pause} \right) \right]$ was filled with zeros, thus extending the pause time.

Cubic spine interpolation gave the first gradient block as shown in Fig. B1 above, and this block was copied to produce the full free waveform.

1. **Single Diffusion Encoding (SDE)**

**
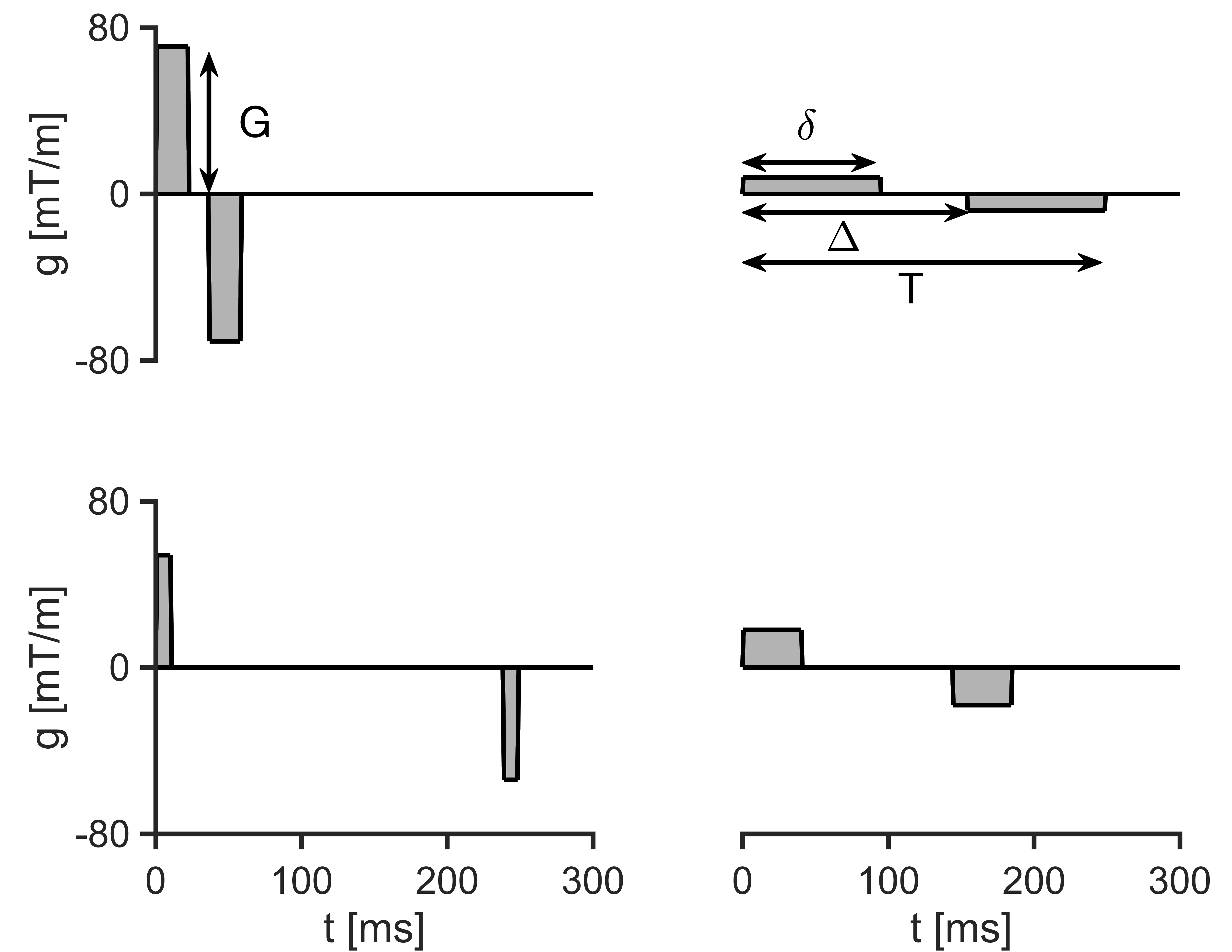
**

Figure A2: Generation of SDE waveforms for probing restriction and exchange. All waveforms shown here have the same b-value of 5 $\mathrm{ms}/\mu m^{2}$.

We generated SDE waveforms by randomly varying the pulse width ($\delta$), the pulse separation ($\Delta$) and the gradient amplitude (G) subject to the following constraints:

1. $\Delta-\delta\geq t_{pause}$ where $t_{pause} = 9$ ms is the minimum pause time between the first and second gradient pulses.
2. $\Delta+ \delta= T \leq200$ms to ensure a maximum encoding time of 200 ms.
3. $G\leq g_{m\mathrm{ax}}$ at a b-value of 0.5 $\mathrm{ms}/\mu m^{2}$ where $g_{m\mathrm{ax}} = 80 mT/m$ is the maximum permissible gradient strength.

For each combination of $\Delta$ and $\delta$, two trapezoidal pulses were created as shown in Fig. B2. The ramp time was fixed for all waveforms and was determined as the ratio between the maximum gradient amplitude ($g_{m\mathrm{ax}}$) and the slew rate (70 T/m/s).

**Appendix B: Monte Carlo simulations**

Random walkers were simulated in 3D space with the aim of generating synthetic signals. The simulation framework was implemented in CUDA C++ and runs on the GPU. Simulation substrates are supplied in the form of a file containing information on the size and location of every cell (cylinder) in the substrate. The simulation world is divided into voxels to accelerate the determination of a particle’s location in the substrate. The voxelisation is similar to that described by Lee et al. ^108^ and is specified by a voxel lookup table and an additional list of indices showing which cell in the substrate, if any, intersects each voxel. Identifying the location of a particle is done by simply taking the integer parts of its x- and y-coordinates and performing a table lookup to determine the cell intersecting the current voxel. A simple computation of the distance between the particle and the centre of that cell is used to determine whether the cell contains the particle. The voxel size is chosen to allow a maximum of one cell to intersect a given voxel. The simulation used periodic boundary conditions with the repeating units depicted in Figure C1. The substrate in (A) consists of hexagonally packed, equally sized parallel cylinders. (B) comprises randomly packed, equally sized parallel cylinders while (C) is composed of parallel cylinders of sizes chosen from a Gamma distribution. The repeating units in (B) and (C) are each made up of at least 1000 cylinders to eliminate variability due to randomness ^109^.


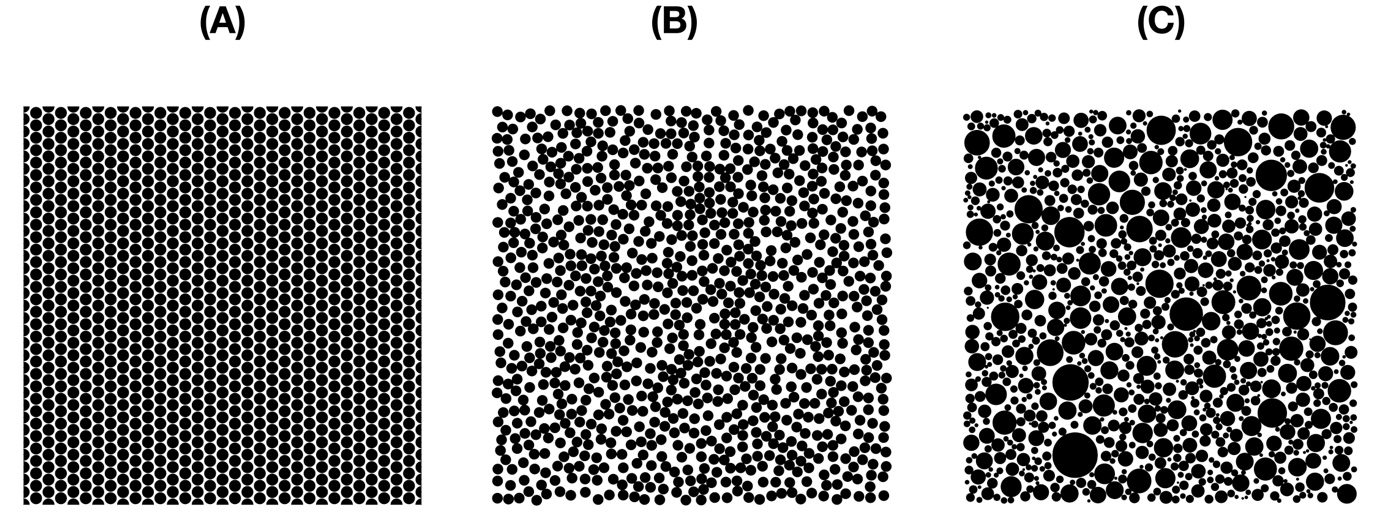


Figure B1: 2D profile through the substrates used in simulations: hexagonally packed cylinders (A), randomly packed cylinders (B) and Gamma-distributed cylinders (C). Darker regions represent the intracellular environment.

Particle steps at every time step had fixed lengths $\Delta r$ and random directions $\hat{\mathbf{n}}$ such that a particle initially at $\mathbf{r}\left( t \right)$ would, after a time $\Delta t$, be found at

$$\begin{aligned} \mathbf{r}\left( t+\Delta t \right)=\mathbf{r}\left( t \right)+\Delta r\cdot\hat{\mathbf{n}}\#\left( B1 \right) \end{aligned}$$

where $\Delta r$ is related to the bulk diffusivity, $D_{0}$*,* through

$$\begin{aligned} \Delta r=\sqrt{2nD_{0}\Delta t}\#\left( B2 \right) \end{aligned}$$

where $n=3$ is the number of dimensions. $D_{0}$ was set to 2 $\mu m^{2}/\mathrm{ms}$ in both compartments. All simulations were performed at a fixed $\Delta t = 1 \mu s$. Particles incident on a membrane crossed it with a probability defined according to ^108,110,111^

$$\begin{aligned} p=4\cdot\kappa\cdot\sqrt{\frac{\Delta t}{2nD_{0}}},\#\left( B3 \right) \end{aligned}$$

where $\kappa$ is the membrane permeability. Particles that did not cross the membrane were returned to their initial positions before the step. The permeability was related to the exchange rate, $k,$ via ^112,113^

$k = \kappa\cdot S/V,$(B4)

where S/V is the surface-to-volume ratio of the cylinder. Simulations were run for exchange rates $k$ between 0 and 20 $s^{-1}$ and diameters $d$ between 1 and 20 µm. Signal fractions were kept fixed at $f_{in}=0.5$ and $f_{ex}=0.5$ where the subscripts *in* and *ex* represent “intracellular” and “extracellular”.

The signal perpendicular to the cylinders was calculated using

$$\begin{aligned} S=\frac{1}{N_{p}}\sum_{n=1}^{N_{p}} \exp\left( -i\phi_{n}\left( T \right) \right),\#\left( B5 \right) \end{aligned}$$

where $N_{p}={10}^{5}$ is the number of particles and $\phi_{n}(T)$ is the particle phase given by

$$\begin{aligned} \phi_{n}\left( T \right)=\gamma\sum_{i=1}^{N} g\left( i \right)\cdot r\left( i \right)\cdot\Delta t,\#\left( B6 \right) \end{aligned}$$

where $N=T/\Delta t$ is the number of time points and $g$ is the gradient waveform.
